# Identification of regions within the *Legionella pneumophila* VipA effector protein involved in actin binding and polymerization and in interference with eukaryotic organelle trafficking

**DOI:** 10.1002/mbo3.316

**Published:** 2015-12-02

**Authors:** Joana N. Bugalhão, Luís Jaime Mota, Irina S. Franco

**Affiliations:** ^1^ UCIBIOREQUIMTEFaculdade de Ciências e TecnologiaDepartamento de Ciências da VidaUniversidade NOVA de LisboaCaparicaPortugal; ^2^ Instituto de Tecnologia Química e Biológica António XavierUniversidade NOVA de LisboaOeirasPortugal

**Keywords:** Actin, effector, *Legionella pneumophila*, type IV secretion system, vesicle trafficking

## Abstract

The *Legionella pneumophila* effector protein VipA is an actin nucleator that co‐localizes with actin filaments and early endosomes in infected macrophages and which interferes with organelle trafficking when expressed in yeast. To identify the regions of VipA involved in its subcellular localization and functions, we ectopically expressed specific VipA mutant proteins in eukaryotic cells. This indicated that the characteristic punctate distribution of VipA depends on its NH_2_‐terminal (amino acid residues 1–133) and central coiled‐coil (amino acid residues 133–206) regions, and suggested a role for the COOH‐terminal (amino acid residues 206–339) region in association with actin filaments and for the NH_2_‐terminal in co‐localization with early endosomes. Co‐immunoprecipitation and in vitro assays showed that the COOH‐terminal region of VipA is necessary and sufficient to mediate actin binding, and is essential but insufficient to induce microfilament formation. Assays in yeast revealed that the NH_2_ and the COOH‐terminal regions, and possibly an NPY motif within the NH_2_ region of VipA, are necessary for interference with organelle trafficking. Overall, this suggests that subversion of eukaryotic vesicular trafficking by VipA involves both its ability to associate with early endosomes via its NH_2_‐terminal region and its capacity to bind and polymerize actin through its COOH‐terminal region.

## Introduction

The gram‐negative pathogen *Legionella pneumophila* is able to invade and replicate inside a diversified group of amoebae. Throughout millions of years of co‐evolution, the bacterium has acquired an array of mechanisms that enable it to infect human alveolar macrophages, causing an atypic pneumonia known as Legionnaires' disease, or the milder illness Pontiac fever. Within host phagocytic cells, Legionellae thrive in a remodeled phagosomal compartment, known as the *Legionella*‐containing vacuole (LCV). Early after cellular uptake, the LCV segregates from the endocytic pathway, and sequentially, recruits vesicles trafficking between the endoplasmic reticulum (ER) and the Golgi, mitochondria and ribosomes, eventually becoming a rough ER‐like compartment. In this modified niche, the bacteria divide until they finally exit the host cell (Franco et al. 2009; Isberg et al. 2009). These processes require a Type IVB secretion system, known as Icm/Dot, which translocates over 300 effector proteins into host cells (Zhu et al. 2011). These are involved in the subversion of key host cell processes such as cytoskeleton dynamics, vesicle trafficking, transcription, translation, lipid signaling, apoptosis, or proteasomal degradation (Hubber et al. 2013; Asrat et al. 2014; Pizarro‐Cerda et al. 2014; Rolando and Buchrieser 2014; Speir et al. 2014).

Microbial pathogens modulate the host cell actin cytoskeleton to control phagocytosis, adhesion, cell–cell contacts, movement within the cytosol, spreading to neighboring cells, or development of a niche viable for intracellular bacterial replication (Kumar and Valdivia 2008; Carabeo 2011; Haglund and Welch 2011). This is accomplished by effector proteins that hijack the major eukaryotic actin nucleators Arp2/3 complex and formins (Truong et al. 2014), modulate key regulators of actin dynamics (e.g., Rho GTPases) (Hardt et al. 1998; Egile et al. 1999; Stender et al. 2000; Zhou et al. 2001; Patel and Galan 2006; Rottner et al. 2010), or directly polymerize actin (Hayward and Koronakis 1999; Jewett et al. 2006; Liverman et al. 2007; Haglund et al. 2010; Franco et al. 2012). The actin network also plays a fundamental role in the *Legionella* infection process. Actin polymerization is necessary for bacterial uptake and subsequent intracellular replication in human macrophages (King et al. 1991). Furthermore, a recent analysis of the human monocyte‐derived macrophages transcriptome upon *L. pneumophila* infection revealed an alteration of expression of host genes encoding proteins involved in cytoskeleton dynamics, such as actin nucleators (Arp2/3 complex subunits, DIA1), nucleation‐promoting factors (WASF1/WAVE1), and other actin‐binding proteins (CapZ, tropomodulin, advillin, alpha‐actinin 4), or Rho GTPases and their effectors (Rac1, RhoA, RhoGAP1, ROCK1, DOCK2) (Price and Abu Kwaik 2014). Similarly, F‐actin formation is also necessary for entry into the amoeba *Dictyostelium*, and several actin‐binding proteins have been shown to favor or counteract *Legionella* entry and intracellular growth, and associate with the LCV (e.g., coronin, cofilin, myosin II, profilin, Arp2/3 components, and actin bundling and capping proteins) (Hagele et al. 2000; Solomon and Isberg 2000; Fajardo et al. 2004; Shevchuk et al. 2009; Urwyler et al. 2009; Peracino et al. 2010; Shina et al. 2010). However, in contrast to other pathogens, the identification and characterization of *Legionella* effectors targeting the actin network has remained elusive. In fact, only three effectors have been implicated in modulating formation of microfilaments, with VipA promoting actin polymerization, and Ceg14 and LegK2 inhibiting it (Franco et al. 2012; Guo et al. 2014; Michard et al. 2015).

**Figure 1:**
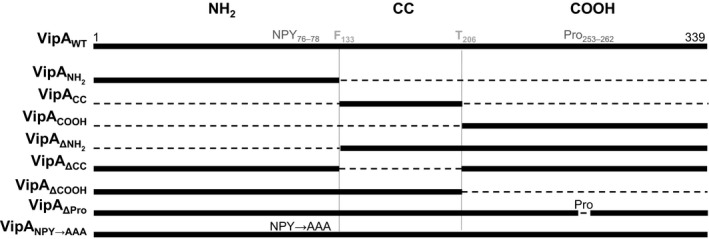
Representation of the deletions and amino acid substitutions introduced in the different VipA mutants studied throughout this work. Numbers correspond to positions of amino acid residues in the primary structure of VipA and dashed lines to deleted regions; NH_2_, NH_2_‐terminal region; CC, coiled‐coil region; COOH, COOH‐terminal region; NPY, asparagine–proline–tyrosine motif; Pro, proline‐rich sequence.

We previously showed that VipA nucleates actin polymerization in vitro, and co‐localizes with actin filaments and early endosomes in infected macrophages (Franco et al. 2012). A similar distribution was observed when VipA was ectopically expressed in mammalian Chinese hamster ovary (CHO) cells or in yeast *Saccharomyces cerevisiae*. In addition, VipA impaired trafficking of yeast vacuolar proteins through the multivesicular body (MVB) pathway, causing vacuolar protein sorting (Vps) defects (Shohdy et al. 2005). To understand how the different regions of VipA (Fig. 1) contribute to its subcellular localization and functions, we analyzed mutant VipA proteins for their subcellular localization in eukaryotic cells, ability to bind and to polymerize actin in vitro, and to induce Vps defects in yeast. The results indicate that the NH_2_ region of VipA (amino acid residues 1 to 133) is necessary for co‐localization with early endosomes, whereas the COOH‐terminal of VipA (amino acid residues 202 to 339) is an actin‐binding region, which is essential, but not sufficient, for VipA‐mediated actin polymerization. We also found that the NH_2_ and COOH‐terminal regions of VipA cooperate to interfere with organelle trafficking in yeast, in a process that might involve the NPY_76–78_ motif of VipA.

## Experimental procedures

### Strains and media

*L. pneumophila* JR32 (Sadosky et al. 1993)*, Escherichia coli*, and *S. cerevisiae* strains (listed in Table S1) used in this work were grown as previously described (Shohdy et al. 2005; Franco et al. 2012).

### Plasmids and oligonucleotides

Plasmids and oligonucleotides used in this study are listed in Tables S2 and S3, as well as details of how relevant plasmids were constructed. For general cloning procedures, restriction enzymes (MBI Fermentas Inc., Burlington, Ontario, Canada), T4 DNA ligase (MBI Fermentas), and Phusion polymerase (Finnzymes Inc., Lafayette, Colorado, USA) were used according to the manufacturer's instructions. The accuracy of the nucleotide sequence in the inserts in all the constructed plasmids was checked by DNA sequencing.

### Mammalian cell culture

Chinese hamster ovary Fc*γ*RII cells (Joiner et al. 1990) were grown in Dulbecco's Modified Eagle Medium (DMEM; Life Technologies, Carlsbad, California, USA) and 10% (v/v) heat‐inactivated fetal bovine serum (FBS; Life Technologies), at 37°C in a 5% (v/v) CO_2_ incubator. CHO cells were transfected using the jetPEI^™^ reagent (Polyplus, Martensville, Hatchettina, Canada) according to manufacturer's protocol.

### Antibodies and fluorescent dyes

The following antibodies were used for immunoblotting: goat anti‐GFP (SICGEN, Cantanhede, Portugal; 1:1000), rabbit anti‐actin (Sigma‐Aldrich, St. Louis, Missouri, USA 1:200), mouse anti‐PGK1 (Life Technologies; 1:1000), mouse anti‐c‐Myc (clone 9E10, Calbiochem, San Diego, California, USA; 1:200), and a rabbit anti‐mCherry (1:1000, a gift from Adriano O. Henriques), followed by anti‐goat, anti‐mouse, or anti‐rabbit horseradish peroxidase (HRP)‐conjugated secondary antibodies (GE Healthcare, Waukesha, Wisconsin, USA or Jackson ImmunoResearch, West Baltimore Pike, West Grove, Pennsylvania, USA 1:10,000). For immunofluorescence microscopy, we used mouse anti‐myc (1:200; Clone 9E10, Calbiochem), and mouse anti‐EEA1 (1:200; BD Transduction Laboratories, San Diego, California, USA) antibodies, followed by appropriate fluorophore‐conjugated anti‐mouse antibodies (Jackson ImmunoResearch; 1:200). Actin staining was carried out by incubating CHO cells with Phalloidin‐Alexa555 (Life Technologies, 1:200) during 30 min.

### Fluorescence microscopy

Transfected CHO cells were fixed and permeabilized for immunofluorescence microscopy as described previously (Franco et al. 2012). Yeast cells were analyzed live, also as previously described (Franco et al. 2012). CHO and yeast cells were analyzed by confocal microscopy on a Laser Scanner Confocal Microscope (Zeiss LSM710, Oberkochen, Germany) and images analyzed with ImageJ 1.45 software. Quantitative analysis of co‐localization of VipA‐EGFP fusion proteins with EEA1 or F‐actin was performed by calculating the Manders overlap coefficient, corresponding to the fraction of green pixels (VipA‐EGFP signal) that overlap with red pixels in relation to the total green pixels. For this purpose, signal intensities for each cell (*n* ≥ 14) were processed in ImageJ and the coefficients determined with the plugin JACoP (Bolte and Cordelieres 2006). Statistical analysis was performed using GraphPad Prism software version 5.02, La Jolla, California, USA *P* values were calculated using one‐way ANOVA and Bonferroni multiple comparison post‐test.

### Immunoblotting

After SDS‐PAGE, the gels were processed for immunoblotting using Trans‐Blot Turbo Transfer System (BioRad, Hercules, California, USA) and 0.2 *μ*m pore‐size nitrocellulose membranes (BioRad). Immunoblot detection was done with Western Lightning ECL Pro (Perkin Elmer, Winter St. Waltham, Massachusetts, USA), and a ChemiDoc XRS + system (BioRad) or exposure to Amersham Hyperfilm ECL (GE Healthcare).

### Preparation of yeast and mammalian cell extracts for immunoblotting

For detection of expression of GFP or mCherry fusion proteins in yeast strains harboring pKS84 or pIF215 derivatives, respectively (de Felipe et al. 2008; Franco et al. 2012), strains were grown on SC‐ura +2% (w/v) fructose plates for 3 days at 30°C, and several colonies were picked and grown for 24 h in identical medium with 2% (w/v) galactose. An amount equivalent to an optical density at 600 nm (OD_600_) of three in a total volume of 40 *μ*L of Laemmli buffer (Tris‐HCl 0.25 mol/L pH 6.8, SDS 10% [w/v], Glycerol 50% [v/v], *β*‐mercaptoethanol 0.5 mol/L, bromophenol blue 0.5% [w/v]), or an OD_600_ of 1 in 15 *μ*L in the case of the strain expressing GFP, was boiled for 5 min and analyzed by immunoblotting.

For detection of EGFP or myc fusion proteins in mammalian cells, transiently transfected CHO seeded on 6‐well plates were washed twice with 1 mL of ice‐cold phosphate‐buffered saline (PBS), scrapped and centrifuged at 500 ***g***, 4°C for 3 min. Pellets containing 8 × 10^5^ cells were resuspended in 50 *μ*L of Laemmli buffer, boiled at 100°C for 5 min, and analyzed by immunoblotting.

### Immunoprecipitation of VipA‐EGFP

Chinese hamster ovary cells were transfected with pEGFP‐N1 and plasmids encoding VipA‐EGFP variants using the jetPEI^™^ transfection reagent (Polyplus) according to manufacturer's protocol. Cells were harvested 24 h post transfection and co‐immunoprecipitations were performed using Chromotek (Hauppauge, New York, USA) GFP‐Trap agarose beads essentially as described by the manufacturer. In brief, cells were lysed with lysis Buffer (50 mmol/L Tris‐HCl pH 8, 150 mmol/L NaCl, 0.1 mmol/L EDTA, 0.5% [w/v] IGEPAL^®^, Company: Sigma‐Aldrich: St. Louis, Missouri, USA CA‐630, 1 mmol/L dithiothreitol [DTT], 100 *μ*g/mL phenylmethylsulfonyl fluoride [PMSF], and a protease Inhibitor cocktail [Amresco]), and the beads were incubated with lysate supernatants for 4 h at 4°C in dilution buffer (50 mmol/L Tris‐HCl pH 8, 150 mmol/L NaCl, 0.1 mmol/L ethylenediaminetetraacetic acid [EDTA], 1 mmol/L DTT, 100 *μ*g/mL PMSF, and a protease Inhibitor cocktail [Amresco]), and washed three times with wash buffer (50 mmol/L Tris‐Hcl pH 8, 150 mmol/L NaCl, 0.1 mmol/L EDTA). Aliquots of input, nonbound, washes, and bound samples were analyzed by immunoblotting.

### Overexpression and purification of His_6_‐VipA proteins

*E. coli* BL21(DE3) strains harboring plasmids encoding 6xHis‐tagged proteins (see Table S1) were grown at 37°C for 18 h (His_6_‐VipA and His_6_‐VipA_ΔNH2_) or at 37°C for 5 h followed by 24 h at 26°C (VipA_ΔCC,_ VipA_ΔCOOH_ and VipA_COOH_) in auto‐induction conditions (as previously described by (Studier 2005). Cells were harvested by centrifugation and the cell pellet resuspended in 10 mL of lysis buffer (50 mmol/L Na_2_HPO_4_, 300 mmol/L NaCl, 20 mmol/L imidazole). Bacteria were lysed with three passages in a French press at 900 Psi in the presence of 1 mmol/L PMSF and lysates were centrifuged at 13,000× ***g*** for 30 min at 4°C. His‐tagged proteins were purified from the soluble fraction using Ni–NTA (nickel–nitrilotriacetic acid) chromatography (Qiagen, Valencia, California, USA) and eluted with Imidazole gradients.

### Yeast Invertase assays

*S. cerevisiae* cells expressing GFP and/or mCherry fusion proteins were grown in plates with YNB‐Ura/‐Leu supplemented with 2% (w/v) fructose at 30°C for 3 days, streaked on the same media with fructose (noninducing media) or galactose (inducing media), grown for 24 h and used for microscopy, invertase assays, or protein extract preparation. Assays for qualitative and quantitative detection of invertase enzymatic activity were performed as described (Darsow et al. 2000; Shohdy et al. 2005).

### In vitro actin polymerization assays

Preparation of G‐Actin and polymerization assays were carried out essentially as described (Franco et al. 2012) with the following alterations. Samples contained 1 *μ*mol/L G‐actin (Cytoskeleton, >99% pure; 10% Pyrene‐actin) and purified His_6_‐VipA mutants in G‐Mg buffer were added as indicated. Polymerization assays were carried out in quartz cuvettes (3 mm optical path length), and fluorescence read in a Varian Cary Eclipse fluorescence reader. Values were obtained using an excitation wavelength of 365 nm and emission of 407 nm (10 nm slit width), and recorded at 1‐min intervals. Data were collected with Cary Eclipse software and then processed in Excel (Microsoft, Redmond, Washington, USA).

## Results

The primary structure of VipA (339 amino acid residues) does not display significant similarities to known proteins or domains. However, VipA has a predicted coiled‐coil region, ranging from a phenylalanine residue at position 133 (F_133_) to a threonine residue at position 206 (T_206_) (Fig. 1), which could be involved in protein–protein interactions. A more thorough examination also revealed two motifs involved in the capacity of several bacterial and mammalian proteins to modulate actin dynamics: an Asparagine–Proline–Tyrosine sequence (residues 76–78; NPY_76–78_) and a region rich in prolines (residues P_253_–P_262_) (Fig. 1) (Reinhard et al. 1995; Gertler et al. 1996; Suetsugu et al. 1998; Ahern‐Djamali et al. 1999; Lambrechts et al. 2000; Brady et al. 2007; He et al. 2009).

To investigate the contribution of the different regions of VipA to its known subcellular localization and functions, we subdivided it in an NH_2_‐terminal region (hereafter named “NH_2_”, from the initial methionine residue to F_133_), a central coiled‐coil region (“CC”, from F_133_ to T_206_), and a COOH‐terminal region (“COOH”, from T_206_ to the terminal leucine residue at position 339) (Fig. 1). We initially constructed eukaryotic expression plasmids encoding VipA deletion mutants containing only one of these regions (VipA_NH2_, VipA_CC_ or VipA_COOH_; Fig. 1) or lacking only one of these regions (VipA_ΔNH2_, VipA_ΔCC_ or VipA_ΔCOOH_; Fig. 1). We also constructed eukaryotic expression plasmids encoding VipA mutant proteins where the NPY_76–78_ motif was replaced by alanines (VipA_NPY→AAA_), or where the proline‐rich region was deleted (VipA_ΔPro_) (Fig. 1).

### Analysis of the localization of ectopically expressed VipA mutant proteins in mammalian cells

**Figure 2:**
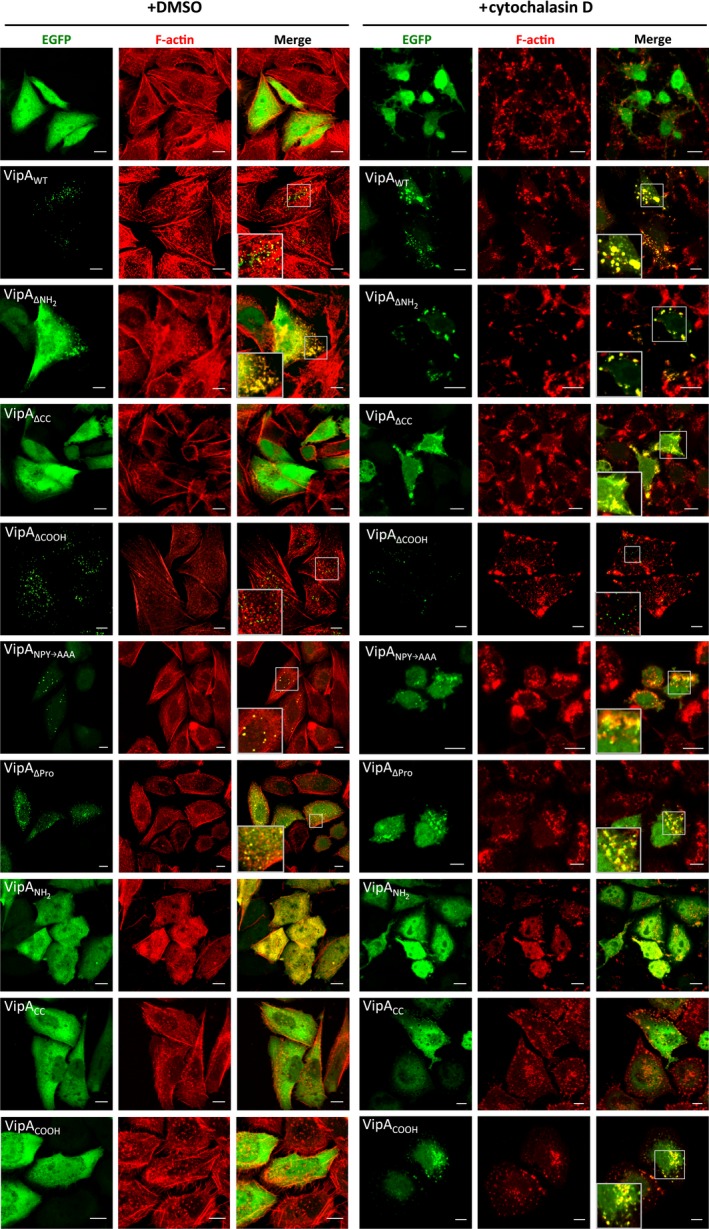
The punctate distribution of VipA in mammalian cells depends on its NH_2_‐terminal and central coiled‐coil regions and the co‐localization of VipA with F‐actin is mediated through its COOH‐terminal region. Cells were transfected with plasmids expressing EGFP or the indicated VipA‐EGFP fusion proteins. After 24 h, cells were incubated with DMSO (left panel) or 10 *μ*mol/L cytochalasin D for 30 min (right panel) immediately before fixation and labeling of F‐actin with phalloidin‐Alexa 555. Images shown are representative and were collected in a Zeiss LSM710 Confocal Microscope. Scale bars, 10 *μ*m.

**Figure 3:**
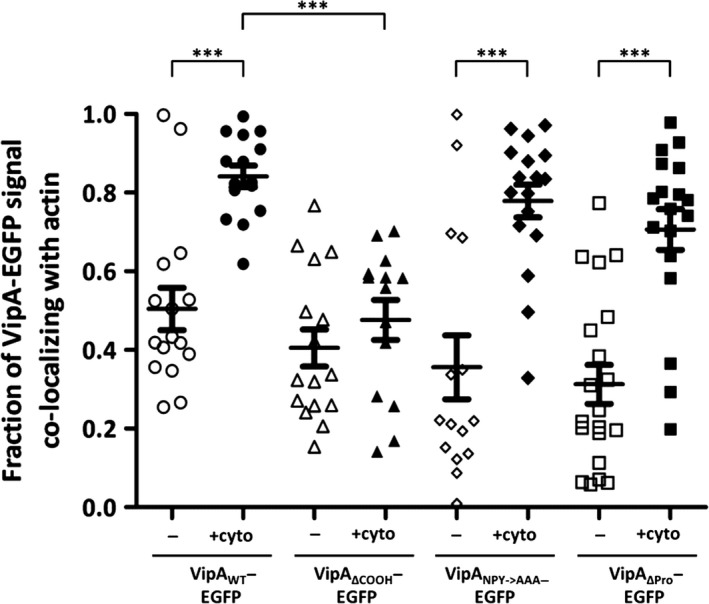
Quantification of co‐localization between VipA‐EGFP fusion proteins and F‐actin in mammalian CHO cells untreated (−) or treated with cytochalasin D (+cyto). Quantification of co‐localization between VipA and actin was performed by calculating the Manders coefficient using ImageJ and the Plugin JACoP (see Experimental procedures). The Manders coefficient corresponds to the fraction of VipA‐EGFP signal (green) overlapping with actin signal (red) divided by the total green signal in a cell. Symbols represent individual cells expressing VipA‐EGFP mutant proteins (*n* ≥ 14 from three independent experiments) and bars indicate mean and standard error of the mean. Statistical analysis was performed as described in Experimental procedures and the obtained *P* value indicating statistically significant differences is indicated (***, *P* < 0.001). All other differences between data sets are not statistically significant (*P* > 0.05).

We first analyzed the distribution of wild‐type VipA (VipA_WT_) or VipA mutant proteins fused to enhanced green fluorescent protein (EGFP) via their C‐termini (VipA‐EGFP proteins) after transient expression in mammalian CHO cells. After 24 h of transfection with plasmids encoding VipA‐EGFP proteins, the cells were fixed, co‐labeled for F‐actin with fluorophore‐conjugated phalloidin, and analyzed by fluorescence microscopy. In a previous work, we had observed a punctate localization of VipA_WT_‐EGFP in CHO cells, a partial co‐localization with F‐actin patches and, to a lesser extent, with early endosomes (Franco et al. 2012). This distribution was also observed for endogenous VipA translocated by *L. pneumophila* during infection of THP‐1 macrophages (Franco et al. 2012), supporting the use of transfected CHO cells as a model for the analysis of the localization of VipA in eukaryotic cells. In the present work, we found that VipA_ΔCOOH_‐EGFP, VipA_NPY→AAA_‐EGFP, and VipA_ΔPro_‐EGFP localized in foci that appear to co‐localize with F‐actin as well as VipA_WT_‐EGFP (Fig. 2, left panel). Interestingly, VipA_ΔNH2_‐EGFP partially localized in patches and elongated structures that markedly overlapped with F‐actin in the subcortical region (Fig. 2; left panel). In contrast, VipA_ΔCC_‐EGFP, VipA_NH2_‐EGFP, VipA_CC_‐EGFP_,_ and VipA_COOH_‐EGFP were homogeneously spread in the cytosol (Fig. 2; left panel). To analyze the co‐localization of VipA_WT_‐EGFP, VipA_ΔCOOH_‐EGFP, VipA_NPY→AAA_‐EGFP, and VipA_ΔPro_‐EGFP with actin objectively, we quantified the proportion of EGFP signal that overlapped phalloidin‐stained actin filaments. This confirmed that when ectopically expressed in CHO cells, these proteins co‐localize with F‐actin in levels ranging from 31% in VipA_ΔPro_‐EGFP to 50% in VipA_WT_‐EGFP, values that shown no statistic difference (Fig. 3).

Analysis of the expression of the VipA‐EGFP proteins by immunoblotting of extracts of transfected CHO cells with an anti‐GFP antibody showed that the fusion proteins migrate on SDS‐PAGE according to their predicted molecular mass, but also revealed several degradation products (Fig. S1). To confirm that the EGFP tag was not affecting protein localization, and that the background fluorescence from the degradation products was not interfering with the analysis, we generated transfection plasmids where EGFP was replaced by a COOH‐terminal myc tag in VipA variants showing a punctate subcellular localization (VipA_WT_, VipA_ΔCOOH_; Fig. 2, left panel), elongated patches that co‐localize with F‐actin (VipA_ΔNH2_; Fig. 2 left panel), or a cytosolic distribution (VipA_ΔCC_; Fig. 2, left panel). These plasmids were used to transfect CHO cells, which after fixation were immunolabeled using anti‐myc antibodies and stained for F‐actin using fluorophore‐conjugated phalloidin. Immunofluorescence microscopy revealed that the localization of these myc‐tagged proteins was similar to the one observed for their counterpart EGFP fusion proteins (Fig. S2A and Fig. 2). Furthermore, immunoblotting of extracts of transfected CHO cells with an anti‐myc antibody revealed that the VipA‐myc proteins migrated on SDS‐PAGE according with their predicted molecular mass and with no detectable degradation products (Fig. S2B).

**Figure 4:**
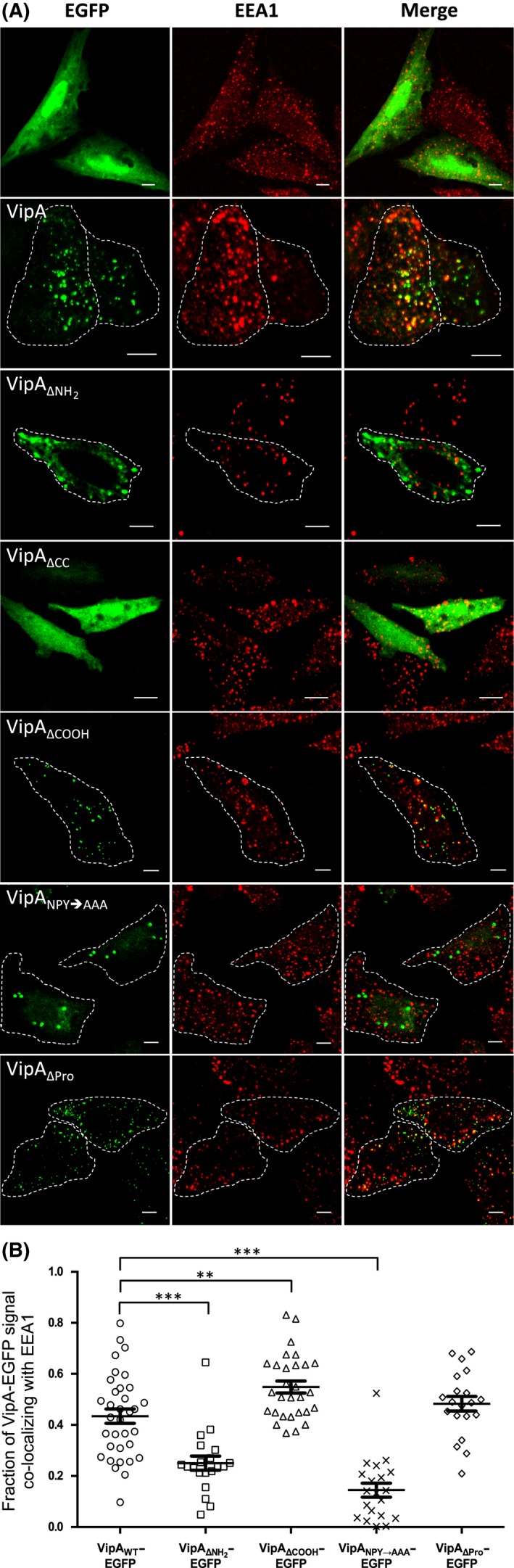
Co‐localization between VipA‐EGFP fusion proteins and EEA1 in mammalian CHO cells. (A) CHO cells were transfected for 24 h with pEGFP‐N1 (Clontech) derivatives encoding the indicated proteins. After transfection, cells were fixed and immunolabeled with an anti‐EEA‐1 antibody and an appropriate fluorophore‐conjugated secondary antibody. Images shown were collected in a Zeiss LSM710 confocal microscope. Scale bars, 5 *μ*m. (B) Quantification of co‐localization between VipA and EEA1 was performed by calculating the Manders coefficient using ImageJ and the Plugin JACoP (see Experimental procedures). The Manders coefficient corresponds to the fraction of VipA‐EGFP signal (green) overlapping with EEA1 signal (red) divided by the total green signal in a cell. Symbols represent individual cells expressing VipA‐EGFP mutant proteins (*n* ≥ 20, from three independent experiments) and bars indicate mean and standard error of the mean. Statistical analysis was performed as described in Experimental Procedures and the obtained *P* values indicating statistically significant differences (*P* < 0.05) are indicated (**, *P* < 0.005; ***, *P* < 0.001).

To analyze the co‐localization of VipA‐EGFP proteins with early endosomes, transfected cells were immunolabeled with an anti‐EEA1 antibody and an appropriate fluorophore‐conjugated antibody and examined by confocal microscopy (Fig. 4A). In each case, several confocal z‐sections were used to quantify the co‐localization of VipA‐EGFP proteins with EEA1 (Fig. 4B). This analysis showed that approximately 43% of VipA_WT_‐EGFP co‐localized with EEA1‐labeled endosomes (Fig. 4B). Relative to VipA_WT_‐EGFP_,_ VipA_ΔPro_‐EGFP (~49%) and VipA_ΔCOOH_‐EGFP (~50%) showed slightly higher levels of co‐localization with EEA1, which in the case of VipA_ΔCOOH_‐EGFP corresponded to a statistically significant difference (Fig. 3B). In contrast, relative to VipA_WT_‐EGFP, VipA_ΔNH2_‐EGFP (~25%) and VipA_NPY→AAA_‐EGFP (~14%) showed an obvious and statistically significant lower co‐localization with EEA‐1‐labeled endosomes (Fig. 4B).

Taken together, the levels of co‐localization of the different VipA proteins with EEA1 in mammalian cells suggested that the NH_2_ region of VipA might be involved in association with early endosomes. The levels of co‐localization between VipA protein and F‐actin were less discriminative likely because of the dense meshwork of microfilaments in CHO cells, which makes the analysis difficult in this case.

### Disruption of microfilaments in CHO cells affects localization of VipA mutant proteins containing the COOH region

It was anticipated that the localization of VipA variants associating with F‐actin would be affected upon disruption of the microfilament network, and reveal a more obvious co‐localization with F‐actin, as previously observed for VipA_WT_ (Franco et al. 2012). To analyze if this was the case for the mutant VipA proteins, we transfected CHO cells with the different plasmids encoding VipA‐EGFP proteins for 24 h and then incubated them with cytochalasin D for 30 min to disrupt the microfilament network. The cells were then fixed and stained for F‐actin with fluorophore‐conjugated phalloidin. After incubation with cytochalasin D, compact stress fibers are disassembled and only smaller cortical F‐actin patches remain (Fig. 2, right panel). In CHO cells expressing VipA_WT_‐EGFP, and as previously reported (Franco et al. 2012), the treatment with cytochalasin D leads to an alteration of VipA_WT_‐EGFP distribution where puncta seem to coalesce and localize in larger patches close to the cell membrane that co‐localize completely with phalloidin‐stained actin (Fig. 2, right panel). Among the VipA proteins that in untreated CHO cells showed a punctate localization similar to VipA_WT_‐EGFP (VipA_ΔCOOH_‐EGFP_,_ VipA_NPY→AAA_‐EGFP and VipA_ΔPro_‐EGFP; Fig. 2, left panel), disruption of actin filaments resulted in the coalescence into actin‐rich patches of those proteins containing the COOH region (VipA_NPY→AAA_‐EGFP and VipA_ΔPro_‐EGFP; Fig. 2, right panel) but not of the mutant protein lacking this region (VipA_ΔCOOH_‐EGFP; Fig. 2, right panel), which remained as small puncta (Fig. 2, right panel). These observations were confirmed by quantifying the degree of co‐localization of these VipA variants with F‐actin (Fig. 3). In cells where F‐actin was disassembled by incubation with cytochalasin D, VipA_WT_‐EGFP, VipA_ΔPro_‐EGFP, and VipA_NPY→AAA_‐EGFP showed similar levels of co‐localization with actin (Fig. 3), which in all cases were significantly higher than those observed in untreated cells (Fig. 3). In contrast, in cells where F‐actin was disassembled, the level of co‐localization of VipA_ΔCOOH_‐EGFP with actin was significantly small than that observed for VipA_WT_‐EGFP, in the same experimental conditions (Fig. 3). Moreover, the levels of co‐localization of VipA_ΔCOOH_‐EGFP with actin were not significantly different between cytochalasin D‐treated cells and untreated cells (Fig. 3). The behavior of myc‐tagged VipA_WT_, VipA_ΔNH2_, VipA_ΔCC_, and VipA_ΔCOOH_ proteins expressed in CHO cells treated with cytochalasin D was identical to that observed in the EGFP counterparts (Fig. 2 and Fig. S2, right panels).

In summary, the analysis of the localization of the different VipA mutant proteins upon cytochalasin treatment indicates that the COOH region of the protein is involved in the co‐localization of the protein with actin and therefore should mediate the interaction of VipA with actin.

### Subcellular localization of VipA proteins in *S. cerevisiae* and co‐localization with actin

In previous work (Franco et al. 2012), VipA‐GFP or VipA‐mCherry expressed in *S. cerevisiae* were shown to appear as foci that co‐localized significantly with yeast actin‐binding proteins (ABPs) present in early endosomes and in cortical actin patches. Therefore, we analyzed the subcellular localization of tagged VipA mutants and their association with ABPs in *S. cerevisiae*. We initially constructed yeast expression plasmids encoding VipA‐GFP fusion proteins, identical to the ones used for expression in CHO cells, under the control of a galactose‐inducible promoter (Table S1). After expression in yeast, VipA_ΔCC_‐GFP, VipA_ΔCOOH_‐GFP, VipA_NPY→AAA_‐GFP, or VipA_ΔPro_‐GFP displayed a punctate distribution similar to the one of VipA_WT_‐GFP (Fig. S3A). Expression of VipA_ΔNH2_‐GFP in yeast also led to the appearance of puncta, but in this case, the foci consistently showed a subcortical localization (Fig. S3A). Similarly to what was observed in CHO cells, expression in yeast cells of VipA_NH2_‐GFP, VipA_CC_‐GFP, or VipA_COOH‐_GFP resulted in their homogeneous distribution in the cytosol (Fig. S3A). The accumulation and migration on SDS‐PAGE with the predicted molecular mass of these proteins was confirmed by immunoblotting of the corresponding cell extracts with an anti‐GFP antibody (Fig. S3B).

**Figure 5:**
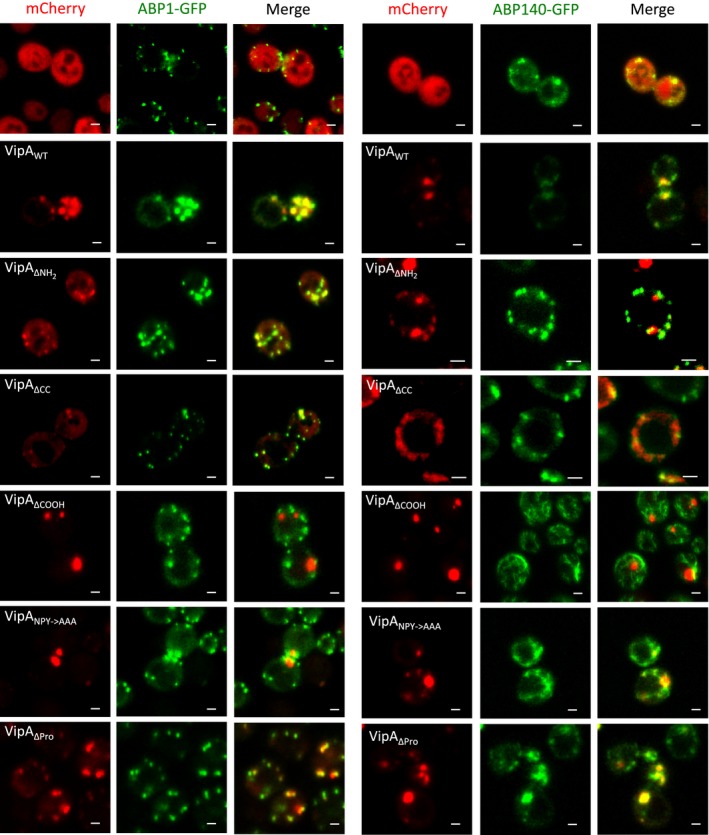
The co‐localization of VipA with actin markers in *Saccharomyces cerevisiae* is mediated by its COOH‐terminal region. Yeast strains producing mCherry or mutant VipA‐mCherry fusion proteins (P_*gal*_‐VipA‐mCherry) and actin markers ABP1‐GFP (left panel) or ABP140‐GFP (right panel) were grown in the presence of galactose and live cells visualized in a Zeiss LSM710 Confocal Microscope. Scale bar, 2 *μ*m.

To assess if, as VipA_WT_, the VipA mutants that appear as foci after their expression in yeast (VipA_ΔNH2_, VipA_ΔCC_, VipA_ΔCOOH_, VipA_NPY→AAA_, VipA_ΔPro_) co‐localize with ABPs, we used two strains constitutively expressing ABP1‐GFP or ABP140‐GFP (markers for actin associated with early endosomes or cortical actin patches/actin cables, respectively) (Huckaba et al. 2004). These strains were used as recipients for plasmids carrying VipA_WT_ or mutant VipA‐mCherry fusions (Franco et al. 2012) (Table S1). The localization of these mCherry‐tagged proteins in yeast was identical to the one observed for the GFP fusions, with all displaying a punctate distribution (Fig. 5). In the case of VipA_WT_, approximately 84% of the VipA‐mCherry foci overlapped with ABP1‐GFP, and ~86% with ABP140‐GFP. These levels were in general similar to the ones seen for VipA_ΔNH2_, VipA_ΔCC_, VipA_NPY→AAA_ and VipA_ΔPro_, whose puncta showed a co‐localization of between 65 and 95% with the actin markers. However, these values were drastically reduced for VipA_ΔCOOH_, where only ~18% of the puncta co‐localized with ABP1‐GFP and ~12% with ABP140‐GFP.

Although ABP1‐GFP and ABP140‐GFP mark distinct actin structures in the yeast cell, these two markers co‐localize themselves during actin patch movement (Huckaba et al. 2004). This explains the similar levels of co‐localization of each VipA protein with ABP1 and ABP140. The presence of fusion proteins and migration on SDS‐PAGE according to the predicted molecular mass was assessed by immunoblot with an anti‐mCherry antibody (Fig. S3C).

In summary, the analyses of the subcellular localization of VipA mutants in yeast indicated that absence of the NH_2_, CC, or COOH regions of VipA does not affect protein localization in foci, while presence of only the NH_2_, CC, or COOH regions leads to a homogenous cytosolic distribution. However, while VipA_WT_, VipA_ΔNH2_ or VipA_ΔCC_ foci significantly co‐localized with ABPs, this was not observed for VipA_ΔCOOH_ foci. Therefore, these results also implicate the COOH region of VipA in mediating the interaction of the protein with actin.

### The COOH region of VipA is necessary and sufficient for actin binding

**Figure 6:**
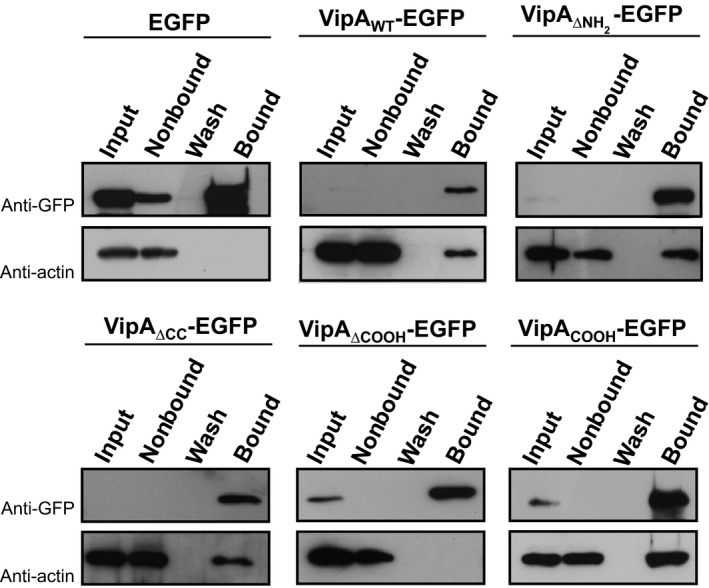
The COOH‐terminal of VipA is an actin‐binding region. Immunoprecipitation assays of VipA‐EGFP fusions were carried out with cell extracts from transfected CHO cells using GFP‐Trap beads (ChromoTek). Immunoblots of the samples corresponding to input fraction (1.5% loaded on gel), nonbound (1.5%), last wash (3%) or bound fractions (60%) were performed with anti‐actin and anti‐GFP antibodies.

To test if the COOH region of VipA indeed mediates binding to actin, we analyzed the interaction of VipA_WT_ with actin by comparison with VipA mutant proteins lacking (VipA_ΔCOOH_) or still carrying the COOH region (VipA_ΔNH2,_ VipA_ΔCC_, and VipA_COOH_)_._ For this, we transfected CHO cells with plasmids expressing EGFP (as control), VipA_WT_‐EGFP, VipA_ΔNH2_‐EGFP_,_ VipA_ΔCC_‐EGFP, VipA_ΔCOOH_‐EGFP_,_ or VipA_COOH_‐EGFP, and immunoprecipitated the fusion proteins from cell lysates using GFP‐Trap beads (Chromotek). Samples corresponding to input, nonbound, wash, and bound fractions were then probed with anti‐GFP and anti‐actin antibodies. As expected, actin was pulled down from a cell lysate containing VipA_WT_‐EGFP, but not from the one that expressed EGFP only (Fig. 6). Furthermore, except for VipA_ΔCOOH_‐EGFP, all VipA‐EGFP mutant proteins analyzed promoted the pull down of actin (Fig. 5). The band corresponding to the VipA_WT_‐EGFP, VipA_ΔNH2_‐EGFP, and VipA_ΔCC_‐EGFP proteins in the input fraction could not be detected because of the low amounts of sample loaded on gel (Fig. 6). To confirm that the position of GFP within VipA was not affecting binding to actin, VipA proteins fused to GFP through their N‐termini were used, and identical results were obtained (data not shown).

Overall, this showed that the COOH region of VipA is necessary and sufficient to pull down endogenous actin from mammalian cells extracts. Because purified VipA can bind actin directly (Franco et al. 2012), this indicates that the COOH region of VipA (Fig. 1) is an actin‐binding region.

### The COOH region of VipA is essential for actin polymerization

**Figure 7:**
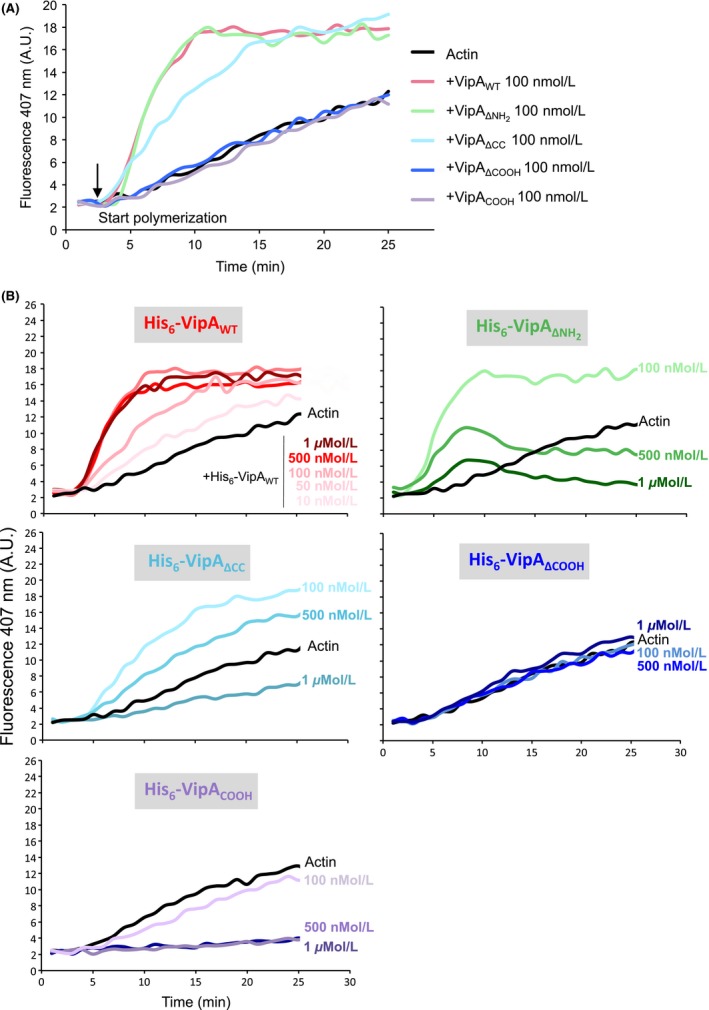
The COOH‐terminal region of VipA is essential, but not sufficient, for VipA‐mediated actin polymerization. In vitro actin polymerization assays were carried out in the presence of 1 *μ*mol/L monomeric actin (10% Pyrene‐actin) and purified wild‐type or mutant His_6_‐VipA. Fluorescence (expressed in Arbitrary Units, AU) was measured over time after initiation of polymerization (at *t* = 3 min). (A) Samples were preincubated with 100 nmol/L of each purified His_6_‐VipA variant. (B) Samples were preincubated with the indicated concentrations of each purified His_6_‐VipA variant. Curves representative of at least three independent experiments are shown.

To test if these mutant proteins were affected in their ability to increase microfilament formation, we carried out in vitro actin polymerization assays. For this, the relevant VipA proteins (VipA_WT_, VipA_ΔNH2_, VipA_ΔCC_, VipA_ΔCOOH_ and VipA_COOH_) were purified as N‐terminally his‐tagged proteins (His_6_‐VipA proteins). We used 1 *μ*mol/L of total G‐actin (10% labeled with pyrene) and tested initially purified His_6_‐VipA proteins at 100 nmol/L, which corresponded to the concentration for which VipA_WT_ presented its highest effect (Franco et al. 2012; Fig. 7A). At this concentration, His_6_‐VipA_ΔNH2_ polymerized actin as well as His_6_‐VipA_WT_, His_6_‐VipA_ΔCC_ retained some ability to polymerize actin, but was not as active as His_6_‐VipA_WT_, and His_6_‐VipA_ΔCOOH_ failed to promote actin polymerization, displaying a curve with levels identical to the reaction containing actin only (Fig. 7A). Interestingly, at 100 nmol/L, VipA_COOH_ also did not influence microfilament formation (Fig. 7A). To see if higher concentrations of His_6_‐VipA proteins had any impact on filament growth rates, we performed identical assays in the presence of increasing concentrations of the purified VipA proteins (Fig. 7B). For His_6_‐VipA_ΔCOOH_, no effect was seen on actin polymerization even with concentrations as high as 1 *μ*mol/L (Fig. 7B). In the cases of His_6_‐VipA_ΔNH2_ and His_6_‐VipA_ΔCC_, higher concentrations led to an inhibition in the formation of microfilaments (Fig. 7B). The impact at high concentrations was drastic in the case of VipA_COOH_, with filament growth being completely blocked (Fig. 7B).

These results showed that the COOH region of VipA is essential, but not sufficient, for the ability of the protein to mediate actin polymerization in vitro. Furthermore, high concentrations of VipA truncated proteins containing the COOH region (VipA_ΔNH2_, VipA_ΔCC_, and VipA_COOH_) inhibit actin polymerization, indicating that both the NH_2_ and the CC regions of VipA play a fundamental role in the mechanism of VipA‐mediated actin polymerization.

### The NH_2_ and COOH regions, and the NPY_76–78_ motif, of VipA are necessary to cause trafficking defects in *S. cerevisiae*

**Figure 8:**
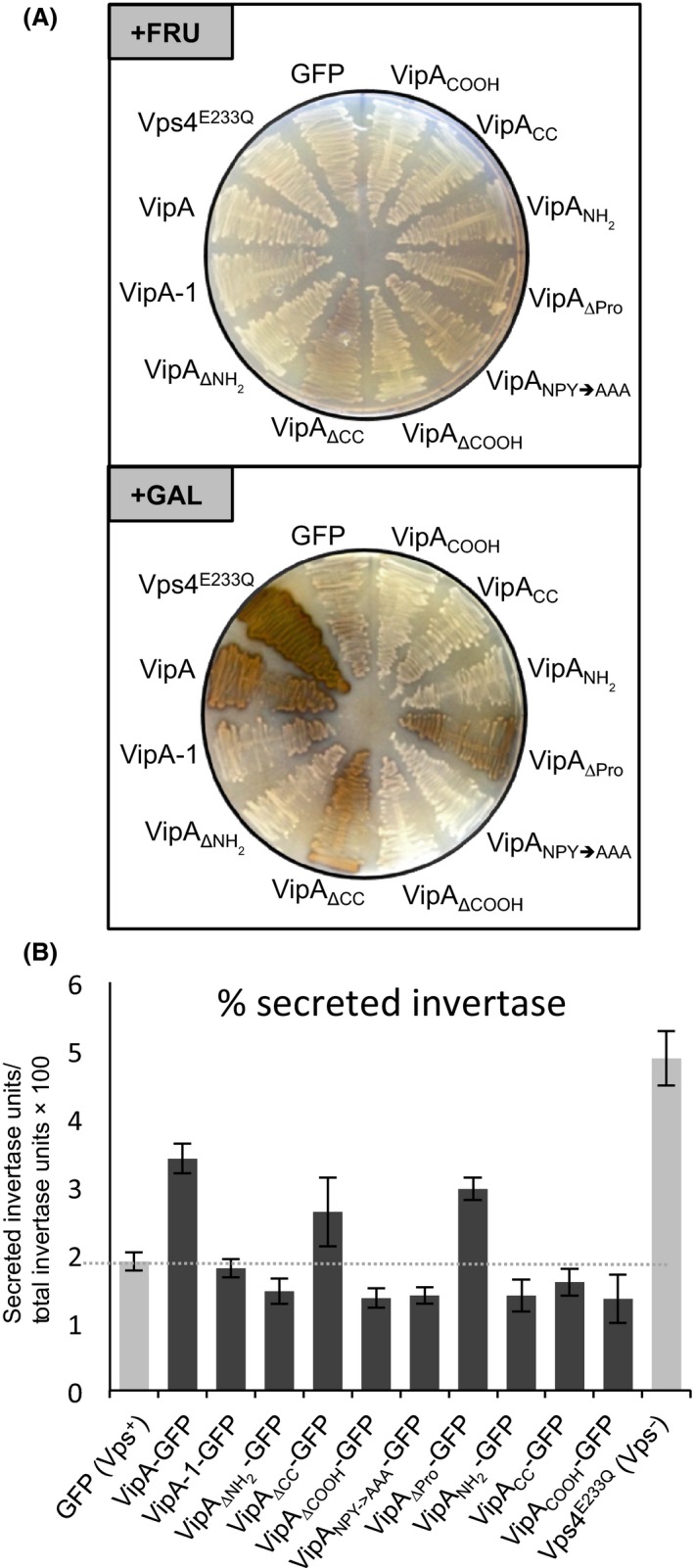
The NH_2_ and COOH terminal regions, and the NPY_76–78_ motif, of VipA are all required for interference with vacuolar protein trafficking in *Saccharomyces cerevisiae*. (A) *S. cerevisiae* NSY01‐derived strains expressing VipA mutants with COOH‐terminal fusions to GFP under the galactose promoter (P_*gal*_‐VipA‐GFP) were grown in inducing (in the presence of galactose; “+GAL”) or noninducing (in the presence of fructose; “+FRU”) conditions. The activity of secreted CPY‐invertase, indicative of defects in vacuolar trafficking pathways, was assessed qualitatively in solid medium using a sucrose overlay assay (see Experimental procedures). (B) The *S. cerevisiae* NSY01‐derived strains were grown in liquid medium in inducing conditions (in the presence of galactose) and the activity of invertase (in the culture supernatant and within yeast cells) was determined quantitatively (see Experimental procedures). The values correspond to the mean and standard error of the mean of at least three independent experiments.

To assess the regions of VipA necessary for its capacity to interfere with organelle trafficking in yeast (Shohdy et al. 2005), we tested the ability of all previously analyzed variants (VipA_ΔNH2_, VipA_ΔCC_, VipA_ΔCOOH,_ VipA_NH2_, VipA_CC_, VipA_COOH_, VipA_NPY→AAA_ and VipA_ΔPro_; Fig. 1) to induce a Vps defect in yeast. For this purpose, we used the *S. cerevisiae* reporter strain NSY01 that expresses a modified form of invertase (CPY‐Inv), which can be diverted from the original vacuolar route to the outside of the cell as a consequence of vacuolar protein mistrafficking. Secreted invertase can then be detected in a colorimetric enzymatic assay qualitatively in solid media, or quantified in liquid media (Darsow et al. 2000; Shohdy et al. 2005). We used the NSY01 strain as recipient to introduce the plasmids carrying the relevant VipA‐GFP fusions. A negative control strain was used that expressed only GFP, giving rise to white colonies in a plate assay (Vps^+^ phenotype), whereas a positive control expressing a dominant negative form of the ATPase Vps4 yielded brown colonies (Vps4^E233Q^, Vps^−^ phenotype; Fig. 8) (Shohdy et al. 2005). A similar Vps^−^ phenotype was observed for a strain expressing VipA_WT_, whereas in strain expressing a VipA mutant analyzed in previous studies, VipA‐1 (Franco et al. 2012) normal vacuolar trafficking is restored (Fig. 8). When compared to VipA_WT_, VipA_ΔCC_ and VipA_ΔPro_ were still able to cause a Vps^−^ phenotype in galactose inducing media, but not in fructose noninducing media (Fig. 8A). However, VipA_ΔNH2_, VipA_ΔCOOH_, VipA_NPY→AAA_, VipA_NH2_, VipA_CC_, and VipA_COOH_ were all unable to cause a Vps^−^ phenotype (Fig. 8A). To get a quantitative measure of this effect, an identical assay was carried out in liquid media where both the amounts of secreted and total invertase were assessed for each strain, with the percentage of secreted enzyme reflecting the intensity of the Vps phenotype (Fig. 8B). Similarly to the qualitative assays, the results obtained indicate that the CC and the proline‐rich regions are not essential for interference of VipA with vacuolar trafficking in yeast (Fig. 8B). In contrast, deletion of the NH_2_ or COOH regions, or substituting the NPY_76–78_ motif for alanines, resulted in an activity of secreted invertase comparable to the negative control strain only expressing GFP (Fig. 8B). This was not due to lower amounts of these proteins in the eukaryotic cell, as ruled out by immunoblot analysis of the corresponding cell extracts (Fig. S3B), which showed that the fusion proteins analyzed accumulate in the cell in amounts similar to VipA_WT_.

Overall, this indicates that the NH_2_ and COOH regions of VipA are both necessary to disrupt vacuolar protein trafficking in *S. cerevisiae*. Furthermore, it suggests a role of the NPY_76–78_ motif within the NH_2_ region of VipA in this process.

## Discussion

The *L. pneumophila* effector collection comprises over 300 proteins, but to date only VipA, Ceg14 and, more recently, LegK2 have been shown to target actin (Franco et al. 2012; Guo et al. 2014; Michard et al. 2015). Ceg14 and LegK2 inhibit actin polymerization, whereas VipA is an actin nucleator that interferes with trafficking in yeast by affecting the MVB pathway (Shohdy et al. 2005). After being translocated into macrophages, VipA localizes to cortical actin patches and in puncta that partially co‐localize with early endosomes, a distribution similar to the one observed when it is ectopically expressed as GFP fusions in mammalian cell lines (Franco et al. 2012). In this work, we analyzed the role of different regions of VipA on its subcelullar localization and known functions. We found that: (1) the NH_2_ and CC regions of VipA are required for its localization in distinct puncta in mammalian cells; (2) the NH_2_ region of VipA, including the NPY_76–78_ motif, is likely involved in co‐localization with early endosomes; (3) the COOH region of VipA is necessary and sufficient for binding to actin and is essential for actin polymerization mediated by VipA; (4) the NH_2_ and COOH regions, including the NPY_76–78_ motif, are required for the protein to interfere with organelle trafficking in yeast. Because VipA mutant proteins that bind and can polymerize actin (VipA_ΔNH2_,) or that do not bind and do not polymerize actin (VipA_ΔCOOH_) are both defective in inducing a Vps defect in yeast, this indicates that binding of VipA to actin and VipA‐mediated actin polymerization are not sufficient for the ability of VipA to interfere with organelle trafficking in yeast. Furthermore, VipA mutant proteins that fail to localize at early endosomes (VipA_ΔNH2_ and VipA_NPY→AAA_) as well as VipA_WT_ are also defective in inducing a Vps defect in yeast. Therefore, our data support a model in which both binding to actin and localization to endosomes are needed for VipA to interfere with eukaryotic vesicular trafficking.

Although actin‐binding assays show the requirement of only the COOH for interaction with VipA, deletion of each major VipA region had an effect on its actin polymerization activity (Fig. 6). Surprisingly and in contrast to the VipA_WT_, mutants that contain the COOH region (VipA_ΔNH2,_ VipA_ΔCC_ and VipA_COOH_) have an inhibitory effect on microfilament formation when present at higher concentrations (e.g., 0.5–1 *μ*mol/L; Fig. 6B). This effect is concentration‐dependent and the kinetics differ between mutants, from a constant inhibition of polymerization observed for VipA_COOH_ and VipA_ΔCC_, to a curve displaying an initial induction of filament growth followed by depolymerization triggered by VipA_ΔNH2_. This biphasic effect of VipA_ΔNH2_ is reminiscent of Formin INF2 (Chhabra and Higgs 2006), that in addition to inducing nucleation and elongation of F‐actin, can also induce depolymerization at the pointed end of microfilaments in a P_i_‐ and profilin‐dependent manner (Chhabra and Higgs 2006). Depolymerization of microfilaments observed for VipA_COOH_ and VipA_ΔCC_ (Fig. 6B) has also been registered for high concentrations of the NH_2_‐terminal of the nucleator Spire, possibly due to the sequestering of actin monomers (Bosch et al. 2007). These results suggest that, although the interaction with actin is mediated by the VipA COOH‐terminal region alone, the other regions are fundamental for its full action on filament growth, possibly by modulating the binding affinity and/or the correct positioning of VipA during its interaction with actin. Further studies are necessary to understand how these VipA variants are affecting actin dynamics and clarify the role played by each protein region in its activity on actin polymerization.

Actin is a key target of intra‐ and extracellular pathogens that may stimulate or block its polymerization. Bacterial effectors have various modes of inducing actin polymerization, either by targeting eukaryotic actin nucleators (Truong et al. 2014), related Nucleation‐Promoting Factors (NPFs) or their cognate upstream Rho GTPase activators (Hardt et al. 1998; Egile et al. 1999; Stender et al. 2000; Zhou et al. 2001; Patel and Galan 2006; Rottner et al. 2010). However, only a few bacterial proteins able to directly polymerize actin per se have been identified to date (reviewed in (Bugalhão et al. 2015). These effector proteins contain a Wiskott–Aldrich homology 2 (WH2) motif, a G‐actin‐binding domain prevalent in actin modulators belonging to the WASP family of NPFs and to the Tandem Monomer Binding family of nucleators such as Spire (Machesky and Insall 1998). Additionally, some contain proline‐rich Formin homology 1 (FH1) domains, which have been assigned diverse roles in the function of these effectors, namely in oligomerization, binding to profilin–actin, and nucleation of actin (Jewett et al. 2006; Tam et al. 2007; Madasu et al. 2013). Although *L. pneumophila* VipA does not contain a consensus sequence for a WH2 domain, its COOH‐terminal region harbors a proline‐rich sequence. However, our results show that this region is not necessary for a punctate localization of VipA in cells, neither for its ability to interfere with yeast vesicle trafficking. In contrast, substitution of NPY_76–78_ for alanines yielded a protein that shows a significant decrease in its co‐localization with EEA‐1 and ability to interfere with yeast organelle trafficking. A role for the NPY motif has been established for the enteropathogenic and enterohemorrhagic *Escherichia coli* (EPEC and EHEC) and *Citrobacter rodentium* effector protein Tir (Kenny 1999; DeVinney et al. 2001; Deng et al. 2003; Brady et al. 2007). Although the mechanisms have not been completely elucidated, this motif is required for pedestal formation by triggering weak actin polymerization, in a process that involves phosphorylation of the tyrosine and recruitment of N‐WASP (in EPEC), and additionally effector EspF_U_ (in EHEC).

In spite of sequence homologies and related mechanistic effects on actin assembly, the function of these bacterial nucleators in the host cell is diversified, from enabling bacterial motility to formation of stress fibers or surface protrusions to promote colonization (Jewett et al. 2006; Liverman et al. 2007; Tam et al. 2007; Haglund et al. 2010; Benanti et al. 2015). Although the specific function of VipA during infection remains unclear, the results point to a distinct role directed at perturbing vesicle trafficking. Interestingly, a function for *Legionella* LegK2 in inhibiting actin polymerization linked to endosomal arrest has been identified recently (Michard et al. 2015). LegK2 localizes to the LCV where it prevents phagolysosomal fusion by blocking local Arp2/3‐mediated actin polymerization in the amoeba *Dictyostelium discoideum*. A similar function in blocking endocytic fusion with the LCV has been assigned to the effector VipD, identified in a screening for *Legionella* genes leading to a Vps^−^ phenotype in yeast at the same time as VipA (Shohdy et al. 2005). VipD is a phospholipase recruited and activated by host GTPases Rab5 and Rab22 to endosomes. In these organelles, VipD contributes to the depletion of PI3P and consequent inability to recruit downstream effectors, thus rendering these compartments fusion incompetent. This process allows nearby located LCVs to bypass the phagosomal compartment in macrophages (Ku et al. 2012; Gaspar and Machner 2014). Thus, the identification and mode of action of *L. pneumophila* effectors that target eukaryotic vesicle trafficking or actin dynamics is coming to light, two functions performed by VipA. Our results are in accordance with a model where this effector may be acting as a link between actin dynamics and the endocytic pathway, with its COOH‐terminal region mediating the interaction with the cytoskeleton component and the NH_2_ region with the endocytic vesicles, possibly altering their lipidic or protein composition. The concerted action of VipA in these two processes may contribute to the *Legionella* avoidance of endolysosomal degradation during infection, or a disturbance in the adaptive immune response, by interfering with antigen presentation.

## Conflict of Interest

None declared.

## Supporting information


**Table S1.** *S. cerevisiae* strains used in this work.
**Table S2.** Plasmids used in this work.
**Table S3.** Oligonucleotides used in this work.
**Figure S1.** Accumulation of VipA‐EGFP fusion proteins in transiently transfected CHO cells.
**Figure S2.** Localization and accumulation of VipA‐myc fusion proteins in mammalian CHO cells.
**Figure S3.** Subcellular localization and accumulation of VipA‐GFP proteins in *S. cerevisiae*.

## References

[mbo3316-bib-0001] Ahern‐Djamali, S. M. , C. Bachmann , P. Hua , S. K. Reddy , A. S. Kastenmeier , U. Walter , et al. 1999 Identification of profilin and src homology 3 domains as binding partners for Drosophila enabled. Proc. Natl Acad. Sci. USA 96:4977–4982. 10220404 10.1073/pnas.96.9.4977 PMC21802

[mbo3316-bib-0002] Asrat, S. , D. A. de Jesus , A. D. Hempstead , V. Ramabhadran , and R. R. Isberg . 2014 Bacterial pathogen manipulation of host membrane trafficking. Annu. Rev. Cell Dev. Biol. 30:79–109. 25103867 10.1146/annurev-cellbio-100913-013439

[mbo3316-bib-0003] Benanti, E. L. , C. M. Nguyen , and M. D. Welch . 2015 Virulent burkholderia species mimic host actin polymerases to drive actin‐based motility. Cell 161:348–360. 25860613 10.1016/j.cell.2015.02.044 PMC4393530

[mbo3316-bib-0004] Bolte, S. , and F. P. Cordelieres . 2006 A guided tour into subcellular colocalization analysis in light microscopy. J. Microsc. 224:213–232. 17210054 10.1111/j.1365-2818.2006.01706.x

[mbo3316-bib-0005] Bosch, M. , K. H. Le , B. Bugyi , J. J. Correia , L. Renault , and M. F. Carlier . 2007 Analysis of the function of Spire in actin assembly and its synergy with formin and profilin. Mol. Cell 28:555–568. 18042452 10.1016/j.molcel.2007.09.018

[mbo3316-bib-0006] Brady, M. J. , K. G. Campellone , M. Ghildiyal , and J. M. Leong . 2007 Enterohaemorrhagic and enteropathogenic *Escherichia coli* Tir proteins trigger a common Nck‐independent actin assembly pathway. Cell. Microbiol. 9:2242–2253. 17521329 10.1111/j.1462-5822.2007.00954.x

[mbo3316-bib-0007] Bugalhão, J. N. , L. J. Mota , and I. S. Franco . 2015 Bacterial nucleators: actin' on actin. Pathog. Dis. pii:ft078. 10.1093/femspd/ftv078 PMC4626583 26416078

[mbo3316-bib-0008] Carabeo, R. 2011 Bacterial subversion of host actin dynamics at the plasma membrane. Cell. Microbiol. 13:1460–1469. 21790944 10.1111/j.1462-5822.2011.01651.x PMC3174476

[mbo3316-bib-0009] Chhabra, E. S. , and H. N. Higgs . 2006 INF2 Is a WASP homology 2 motif‐containing formin that severs actin filaments and accelerates both polymerization and depolymerization. J. Biol. Chem. 281:26754–26767. 16818491 10.1074/jbc.M604666200

[mbo3316-bib-0010] Darsow, T. , G. Odorizzi , and S. D. Emr . 2000 Invertase fusion proteins for analysis of protein trafficking in yeast. Methods Enzymol. 327:95–106. 11044977 10.1016/s0076-6879(00)27270-4

[mbo3316-bib-0011] Deng, W. , B. A. Vallance , Y. Li , J. L. Puente , and B. B. Finlay . 2003 Citrobacter rodentium translocated intimin receptor (Tir) is an essential virulence factor needed for actin condensation, intestinal colonization and colonic hyperplasia in mice. Mol. Microbiol. 48:95–115. 12657048 10.1046/j.1365-2958.2003.03429.x

[mbo3316-bib-0012] DeVinney, R. , J. L. Puente , A. Gauthier , D. Goosney , and B. B. Finlay . 2001 Enterohaemorrhagic and enteropathogenic *Escherichia coli* use a different Tir‐based mechanism for pedestal formation. Mol. Microbiol. 41:1445–1458. 11580847 10.1046/j.1365-2958.2001.02617.x

[mbo3316-bib-0013] Egile, C. , T. P. Loisel , V. Laurent , R. Li , D. Pantaloni , P. J. Sansonetti , et al. 1999 Activation of the CDC42 effector N‐WASP by the *Shigella flexneri* IcsA protein promotes actin nucleation by Arp2/3 complex and bacterial actin‐based motility. J. Cell Biol. 146:1319–1332. 10491394 10.1083/jcb.146.6.1319 PMC2156126

[mbo3316-bib-0014] Fajardo, M. , M. Schleicher , A. Noegel , S. Bozzaro , S. Killinger , K. Heuner , et al. 2004 Calnexin, calreticulin and cytoskeleton‐associated proteins modulate uptake and growth of *Legionella pneumophila* in *Dictyostelium discoideum* . Microbiology 150:2825–2835. 15347742 10.1099/mic.0.27111-0

[mbo3316-bib-0015] de Felipe, K. S. , R. T. Glover , X. Charpentier , O. R. Anderson , M. Reyes , C. D. Pericone , et al. 2008 Legionella eukaryotic‐like type IV substrates interfere with organelle trafficking. PLoS Pathog. 4:e1000117. 18670632 10.1371/journal.ppat.1000117 PMC2475511

[mbo3316-bib-0016] Franco, I. S. , H. A. Shuman , and X. Charpentier . 2009 The perplexing functions and surprising origins of *Legionella pneumophila* type IV secretion effectors. Cell. Microbiol. 11:1435–1443. 19563462 10.1111/j.1462-5822.2009.01351.x

[mbo3316-bib-0017] Franco, I. S. , N. Shohdy , and H. A. Shuman . 2012 The Legionella pneumophila effector VipA is an actin nucleator that alters host cell organelle trafficking. PLoS Pathog. 8:e1002546. 22383880 10.1371/journal.ppat.1002546 PMC3285593

[mbo3316-bib-0018] Gaspar, A. H. , and M. P. Machner . 2014 VipD is a Rab5‐activated phospholipase A1 that protects *Legionella pneumophila* from endosomal fusion. Proc. Natl Acad. Sci. USA 111:4560–4565. 24616501 10.1073/pnas.1316376111 PMC3970493

[mbo3316-bib-0019] Gertler, F. B. , K. Niebuhr , M. Reinhard , J. Wehland , and P. Soriano . 1996 Mena, a relative of VASP and Drosophila Enabled, is implicated in the control of microfilament dynamics. Cell 87:227–239. 8861907 10.1016/s0092-8674(00)81341-0

[mbo3316-bib-0020] Guo, Z. , R. Stephenson , J. Qiu , S. Zheng , and Z. Q. Luo . 2014 A Legionella effector modulates host cytoskeletal structure by inhibiting actin polymerization. Microbes Infect. 16:225–236. 24286927 10.1016/j.micinf.2013.11.007 PMC3965633

[mbo3316-bib-0021] Hagele, S. , R. Kohler , H. Merkert , M. Schleicher , J. Hacker , and M. Steinert . 2000 Dictyostelium discoideum: a new host model system for intracellular pathogens of the genus *Legionella* . Cell. Microbiol. 2:165–171. 11207573 10.1046/j.1462-5822.2000.00044.x

[mbo3316-bib-0022] Haglund, C. M. , and M. D. Welch . 2011 Pathogens and polymers: microbe‐host interactions illuminate the cytoskeleton. J. Cell Biol. 195:7–17. 21969466 10.1083/jcb.201103148 PMC3187711

[mbo3316-bib-0023] Haglund, C. M. , J. E. Choe , C. T. Skau , D. R. Kovar , and M. D. Welch . 2010 Rickettsia Sca2 is a bacterial formin‐like mediator of actin‐based motility. Nat. Cell Biol. 12:1057–1063. 20972427 10.1038/ncb2109 PMC3136050

[mbo3316-bib-0024] Hardt, W. D. , L. M. Chen , K. E. Schuebel , X. R. Bustelo , and J. E. Galan . 1998 *S. typhimurium* encodes an activator of Rho GTPases that induces membrane ruffling and nuclear responses in host cells. Cell 93:815–826. 9630225 10.1016/s0092-8674(00)81442-7

[mbo3316-bib-0025] Hayward, R. D. , and V. Koronakis . 1999 Direct nucleation and bundling of actin by the SipC protein of invasive Salmonella. EMBO J. 18:4926–4934. 10487745 10.1093/emboj/18.18.4926 PMC1171564

[mbo3316-bib-0026] He, H. J. , X. S. Wang , R. Pan , D. L. Wang , M. N. Liu , and R. Q. He . 2009 The proline‐rich domain of tau plays a role in interactions with actin. BMC Cell Biol 10:81. 19895707 10.1186/1471-2121-10-81 PMC2784441

[mbo3316-bib-0027] Hubber, A. , T. Kubori , and H. Nagai . 2013 Modulation of the ubiquitination machinery by Legionella. Curr. Top. Microbiol. Immunol. 376:227–247. 23918174 10.1007/82_2013_343

[mbo3316-bib-0028] Huckaba, T. M. , A. C. Gay , L. F. Pantalena , H. C. Yang , and L. A. Pon . 2004 Live cell imaging of the assembly, disassembly, and actin cable‐dependent movement of endosomes and actin patches in the budding yeast, *Saccharomyces cerevisiae* . J. Cell Biol. 167:519–530. 15534003 10.1083/jcb.200404173 PMC2172478

[mbo3316-bib-0029] Isberg, R. R. , T. J. O'Connor , and M. Heidtman . 2009 The Legionella pneumophila replication vacuole: making a cosy niche inside host cells. Nature reviews. Microbiology 7:13–24. 19011659 10.1038/nrmicro1967 PMC2631402

[mbo3316-bib-0030] Jewett, T. J. , E. R. Fischer , D. J. Mead , and T. Hackstadt . 2006 Chlamydial TARP is a bacterial nucleator of actin. Proc. Natl Acad. Sci. USA 103:15599–15604. 17028176 10.1073/pnas.0603044103 PMC1622868

[mbo3316-bib-0031] Joiner, K. A. , S. A. Fuhrman , H. M. Miettinen , L. H. Kasper , and I. Mellman . 1990 Toxoplasma gondii: fusion competence of parasitophorous vacuoles in Fc receptor‐transfected fibroblasts. Science 249:641–646. 2200126 10.1126/science.2200126

[mbo3316-bib-0032] Kenny, B. 1999 Phosphorylation of tyrosine 474 of the enteropathogenic *Escherichia coli* (EPEC) Tir receptor molecule is essential for actin nucleating activity and is preceded by additional host modifications. Mol. Microbiol. 31:1229–1241. 10096089 10.1046/j.1365-2958.1999.01265.x

[mbo3316-bib-0033] King, C. H. , B. S. Fields , E. B. Jr Shotts , and E. H. White . 1991 Effects of cytochalasin D and methylamine on intracellular growth of Legionella pneumophila in amoebae and human monocyte‐like cells. Infect. Immun. 59:758–763. 1997428 10.1128/iai.59.3.758-763.1991 PMC258324

[mbo3316-bib-0034] Ku, B. , K. H. Lee , W. S. Park , C. S. Yang , J. Ge , S. G. Lee , et al. 2012 VipD of Legionella pneumophila targets activated Rab5 and Rab22 to interfere with endosomal trafficking in macrophages. PLoS Pathog. 8:e1003082. 23271971 10.1371/journal.ppat.1003082 PMC3521694

[mbo3316-bib-0035] Kumar, Y. , and R. H. Valdivia . 2008 Reorganization of the host cytoskeleton by the intracellular pathogen *Chlamydia trachomatis* . Commun. Integr. Biol. 1:175–177. 19704885 10.4161/cib.1.2.7146 PMC2686014

[mbo3316-bib-0036] Lambrechts, A. , A. V. Kwiatkowski , L. M. Lanier , J. E. Bear , J. Vandekerckhove , C. Ampe , et al. 2000 cAMP‐dependent protein kinase phosphorylation of EVL, a Mena/VASP relative, regulates its interaction with actin and SH3 domains. J. Biol. Chem. 275:36143–36151. 10945997 10.1074/jbc.M006274200

[mbo3316-bib-0037] Liverman, A. D. , H. C. Cheng , J. E. Trosky , D. W. Leung , M. L. Yarbrough , D. L. Burdette , et al. 2007 Arp2/3‐independent assembly of actin by Vibrio type III effector VopL. Proc. Natl Acad. Sci. USA 104:17117–17122. 17942696 10.1073/pnas.0703196104 PMC2040399

[mbo3316-bib-0038] Machesky, L. M. , and R. H. Insall . 1998 Scar1 and the related Wiskott‐Aldrich syndrome protein, WASP, regulate the actin cytoskeleton through the Arp2/3 complex. Curr. Biol. 8:1347–1356. 9889097 10.1016/s0960-9822(98)00015-3

[mbo3316-bib-0039] Madasu, Y. , C. Suarez , D. J. Kast , D. R. Kovar , and R. Dominguez . 2013 Rickettsia Sca2 has evolved formin‐like activity through a different molecular mechanism. Proc. Natl Acad. Sci. USA 110:E2677–E2686. 23818602 10.1073/pnas.1307235110 PMC3718132

[mbo3316-bib-0040] Michard, C. , D. Sperandio , N. Bailo , J. Pizarro‐Cerda , L. LeClaire , E. Chadeau‐Argaud , et al. 2015 The legionella kinase LegK2 targets the ARP2/3 complex to inhibit actin nucleation on phagosomes and allow bacterial evasion of the late endocytic pathway. mBio 6:e00354–15. 25944859 10.1128/mBio.00354-15 PMC4436068

[mbo3316-bib-0041] Patel, J. C. , and J. E. Galan . 2006 Differential activation and function of Rho GTPases during Salmonella‐host cell interactions. J. Cell Biol. 175:453–463. 17074883 10.1083/jcb.200605144 PMC2064522

[mbo3316-bib-0042] Peracino, B. , A. Balest , and S. Bozzaro . 2010 Phosphoinositides differentially regulate bacterial uptake and Nramp1‐induced resistance to Legionella infection in Dictyostelium. J. Cell Sci. 123:4039–4051. 21045112 10.1242/jcs.072124

[mbo3316-bib-0043] Pizarro‐Cerda, J. , A. Kuhbacher , and P. Cossart . 2014 Phosphoinositides and host‐pathogen interactions. Biochim. Biophys. Acta 6:911–918. 25241942 10.1016/j.bbalip.2014.09.011

[mbo3316-bib-0044] Price, C. T. , and Y. Abu Kwaik . 2014 The transcriptome of Legionella pneumophila‐infected human monocyte‐derived macrophages. PLoS ONE 9:e114914. 25485627 10.1371/journal.pone.0114914 PMC4259488

[mbo3316-bib-0045] Reinhard, M. , K. Jouvenal , D. Tripier , and U. Walter . 1995 Identification, purification, and characterization of a zyxin‐related protein that binds the focal adhesion and microfilament protein VASP (vasodilator‐stimulated phosphoprotein). Proc. Natl Acad. Sci. USA 92:7956–7960. 7644520 10.1073/pnas.92.17.7956 PMC41265

[mbo3316-bib-0046] Rolando, M. , and C. Buchrieser . 2014 Legionella pneumophila type IV effectors hijack the transcription and translation machinery of the host cell. Trends Cell Biol. 24:771–778. 25012125 10.1016/j.tcb.2014.06.002

[mbo3316-bib-0047] Rottner, K. , J. Hanisch , and K. G. Campellone . 2010 WASH, WHAMM and JMY: regulation of Arp2/3 complex and beyond. Trends Cell Biol. 20:650–661. 20888769 10.1016/j.tcb.2010.08.014

[mbo3316-bib-0048] Sadosky, A. B. , L. A. Wiater , and H. A. Shuman . 1993 Identification of Legionella pneumophila genes required for growth within and killing of human macrophages. Infect. Immun. 61:5361–5373. 8225610 10.1128/iai.61.12.5361-5373.1993 PMC281323

[mbo3316-bib-0049] Shevchuk, O. , C. Batzilla , S. Hagele , H. Kusch , S. Engelmann , M. Hecker , et al. 2009 Proteomic analysis of Legionella‐containing phagosomes isolated from Dictyostelium. Int. J. Med. Microbiol. 299:489–508. 19482547 10.1016/j.ijmm.2009.03.006

[mbo3316-bib-0050] Shina, M. C. , C. Unal , L. Eichinger , A. Muller‐Taubenberger , M. Schleicher , M. Steinert , et al. 2010 A Coronin7 homolog with functions in actin‐driven processes. J. Biol. Chem. 285:9249–9261. 20071332 10.1074/jbc.M109.083725 PMC2838343

[mbo3316-bib-0051] Shohdy, N. , J. A. Efe , S. D. Emr , and H. A. Shuman . 2005 Pathogen effector protein screening in yeast identifies Legionella factors that interfere with membrane trafficking. Proc. Natl Acad. Sci. USA 102:4866–4871. 15781869 10.1073/pnas.0501315102 PMC555709

[mbo3316-bib-0052] Solomon, J. M. , and R. R. Isberg . 2000 Growth of *Legionella pneumophila* in *Dictyostelium discoideum*: a novel system for genetic analysis of host‐pathogen interactions. Trends Microbiol. 8:478–480. 11044684 10.1016/s0966-842x(00)01852-7

[mbo3316-bib-0053] Speir, M. , J. E. Vince , and T. Naderer . 2014 Programmed cell death in Legionella infection. Future Microbiol. 9:107–118. 24328384 10.2217/fmb.13.139

[mbo3316-bib-0054] Stender, S. , A. Friebel , S. Linder , M. Rohde , S. Mirold , and W. D. Hardt . 2000 Identification of SopE2 from *Salmonella typhimurium*, a conserved guanine nucleotide exchange factor for Cdc42 of the host cell. Mol. Microbiol. 36:1206–1221. 10931274 10.1046/j.1365-2958.2000.01933.x

[mbo3316-bib-0055] Studier, F. W. 2005 Protein production by auto‐induction in high density shaking cultures. Protein Expr. Purif. 41:207–234. 15915565 10.1016/j.pep.2005.01.016

[mbo3316-bib-0056] Suetsugu, S. , H. Miki , and T. Takenawa . 1998 The essential role of profilin in the assembly of actin for microspike formation. EMBO J. 17:6516–6526. 9822597 10.1093/emboj/17.22.6516 PMC1170999

[mbo3316-bib-0057] Tam, V. C. , D. Serruto , M. Dziejman , W. Brieher , and J. J. Mekalanos . 2007 A type III secretion system in Vibrio cholerae translocates a formin/spire hybrid‐like actin nucleator to promote intestinal colonization. Cell Host Microbe 1:95–107. 18005688 10.1016/j.chom.2007.03.005

[mbo3316-bib-0058] Truong, D. , J. W. Copeland , and J. H. Brumell . 2014 Bacterial subversion of host cytoskeletal machinery: hijacking formins and the Arp2/3 complex. BioEssays 36:687–696. 24849003 10.1002/bies.201400038

[mbo3316-bib-0059] Urwyler, S. , Y. Nyfeler , C. Ragaz , H. Lee , L. N. Mueller , R. Aebersold , et al. 2009 Proteome analysis of Legionella vacuoles purified by magnetic immunoseparation reveals secretory and endosomal GTPases. Traffic 10:76–87. 18980612 10.1111/j.1600-0854.2008.00851.x

[mbo3316-bib-0060] Zhou, D. , L. M. Chen , L. Hernandez , S. B. Shears , and J. E. Galan . 2001 A Salmonella inositol polyphosphatase acts in conjunction with other bacterial effectors to promote host cell actin cytoskeleton rearrangements and bacterial internalization. Mol. Microbiol. 39:248–259. 11136447 10.1046/j.1365-2958.2001.02230.x

[mbo3316-bib-0061] Zhu, W. , S. Banga , Y. Tan , C. Zheng , R. Stephenson , J. Gately , et al. 2011 Comprehensive identification of protein substrates of the Dot/Icm type IV transporter of *Legionella pneumophila* . PLoS ONE 6:e17638. 21408005 10.1371/journal.pone.0017638 PMC3052360

