# Supplementary material for: Identification of regions within the Legionella pneumophila VipA effector protein involved in actin binding and polymerization and in interference with eukaryotic organelle trafficking
Source: Microbiologyopen. 2015 Dec 2;5(1):118–33. doi: 10.1002/mbo3.316 (PMC4767423; doi:10.1002/mbo3.316)
Supplement: Supplementary file 1 — Table S1. S. cerevisiae strains used in this work. Table S2. Plasmids used in this work. Table S3. Oligonucleotides used in this work. Figure S1. Accumulation of VipA‐EGFP fusion proteins in transiently transfected CHO cells. Figure S2. Localization and accumulation of VipA‐myc fusion proteins in mammalian CHO cells. Figure S3. Subcellular localization and accumulation of VipA‐GFP proteins in S. cerevisiae. [file MBO3-5-118-s001.docx]

**Supplemental material**

**Identification of regions within the *Legionella pneumophila* VipA effector protein involved in actin binding and polymerization and in interference with eukaryotic organelle trafficking**

Joana N. Bugalhão^1^, Luís Jaime Mota^1,2^, and Irina S. Franco*^1,2^

^1^UCIBIO, REQUIMTE, Departamento de Ciências da Vida, Faculdade de Ciências e Tecnologia, Universidade Nova de Lisboa, Caparica, Portugal.

^2^Instituto de Tecnologia Química e Biológica António Xavier, Universidade Nova de Lisboa, Oeiras, Portugal.

* address correspondence to Irina S. Franco, irinafranco@fct.unl.pt

Running Head: Interaction of *L. pneumophila* VipA with actin

Table S1. *S. cerevisiae* strains used in this work

| **Strain** | **Relevant genotype** | **Construction** | **Reference** |
| --- | --- | --- | --- |
| NSY01 | BHY10 diploid a/α, CPY-Inv, inv-, ura- |  | ([Shohdy *et al.*, 2005](#_ENREF_52)) |
| SCIF00 | NSY01 *Pgal-gfp* | pKS84 → NSY01 | ([Franco *et al.*, 2012](#_ENREF_15) |
| SCIF01 | NSY01 *Pgal-vipA-gfp* | pIF206 → NSY01 | ([Franco *et al.*, 2012](#_ENREF_15)) |
| SCIF50 | *Pgal-vipA_∆NH2_-gfp* BHY10 a/alpha, CPY-Inv, inv-, ura+ | pIF308 → NSY01 | this work |
| SCIF51 | *Pgal-vipA_NPY->AAA_-gfp* BHY10 a/alpha, CPY-Inv, inv-, ura+ | pIF309 → NSY01 | this work |
| SCIF52 | *Pgal-vipA_∆CC_-gfp* BHY10 a/alpha, CPY-Inv, inv-, ura+ | pIF310 → NSY01 | this work |
| SCIF53 | *Pgal-vipA_∆Pro_-gfp*  BHY10 a/alpha, CPY-Inv, inv-, ura+ | pIF311 → NSY01 | this work |
| SCIF58 | *Pgal-vipA_NH2_-gfp* BHY10 a/alpha, CPY-Inv, inv-, ura+ | pIF323 → NSY01 | this work |
| SCIF59 | *Pgal-vipA_CC_-gfp* BHY10 a/alpha, CPY-Inv, inv-, ura+ | pIF324 → NSY01 | this work |
| SCIF60 | *Pgal-vipA_COOH_-gfp* BHY10 a/alpha, CPY-Inv, inv-, ura+ | pIF325 → NSY01 | this work |
| SCIF75 | *Pgal-vipA_∆COOH_-gfp* BHY10 a/alpha, CPY-Inv, inv-, ura+ | pIF327 → NSY01 | this work |
| THY157 | MATa *his3Δ1 leu2Δ0 met15Δ0 ura3Δ0 abp1:gfp:kanMX6* |  | ([Huckaba *et al.*, 2004](#_ENREF_27)) |
| SCIF29 | *abp1-gfp Pgal-mCherry* | pIF215 → THY157 | ([Franco *et al.*, 2012](#_ENREF_15)) |
| SCIF30 | *abp1-gfp Pgal-vipA-mCherry* | pIF216 → THY157 | ([Franco *et al.*, 2012](#_ENREF_15)) |
| SCIF54 | *abp1-gfp Pgal-vipA_∆NH2_-mCherry* | pIF318→ THY157 | this work |
| SCIF55 | *abp1-gfp Pgal-vipA_∆CC_-mCherry* | pIF319→ THY157 | this work |
| SCIF61 | *abp1-gfp Pgal-vipA_NH2_-mCherry* | pIF331→ THY157 | this work |
| SCIF62 | *abp1-gfp Pgal-vipA_CC_-mCherry* | pIF332→ THY157 | this work |
| SCIF63 | *abp1-gfp Pgal-vipA_COOH_-mCherry* | pIF333→ THY157 | this work |
| SCIF67 | *abp1-gfp Pgal-vipA_∆COOH_-mCherry* | pIF330→ THY157 | this work |
| SCJB01 | *abp1-gfp Pgal-vipA_NPY->AAA_-mCherry* | pJB3→ THY157 | this work |
| SCJB02 | *abp1-gfp Pgal-vipA_∆Pro_-mCherry* | pJB4→ THY157 | this work |
| YCY027 | MATa *his3Δ1 leu2Δ0 met15Δ0 ura3Δ0 abp140:gfp:kanMX6* |  | ([Huckaba *et al.*, 2004](#_ENREF_27)) |
| SCIF32 | *abp140-gfp Pgal-mCherry* | pIF215 → YCY027 | ([Franco *et al.*, 2012](#_ENREF_15)) |
| SCIF33 | *abp140-gfp Pgal-vipA-mCherry* | pIF216 → YCY027 | ([Franco *et al.*, 2012](#_ENREF_15)) |
| SCIF56 | *abp140-gfp Pgal-vipA_∆NH2_-mCherry* | pIF318 → YCY027 | this work |
| SCIF57 | *abp140-gfp Pgal-vipA_∆CC_-mCherry* | pIF319 → YCY027 | this work |
| SCIF64 | *abp140-gfp Pgal-vipA_NH2_-mCherry* | pIF331 → YCY027 | this work |
| SCIF65 | *abp140-gfp Pgal-vipA_CC_-mCherry* | pIF332 → YCY027 | this work |
| SCIF66 | *abp140-gfp Pgal-vipA_COOH_-mCherry* | pIF333 → YCY027 | this work |
| SCIF68 | *abp140-gfp Pgal-vipA_∆COOH_-mCherry* | pIF330 → YCY027 | this work |
| SCJB03 | *abp140-gfp Pgal-vipA_NPY->AAA_-mCherry* | pJB3 → YCY027 | this work |
| SCJB04 | *abp140-gfp Pgal-vipA_∆Pro_-mCherry* | pJB4 → YCY027 | this work |

Table S2. Plasmids used in this work

| **Plasmid** | **Purpose/Construction** | **Reference** |
| --- | --- | --- |
| **pGEM-T derivatives** | **Template for further cloning of different *vipA* alleles** | Promega |
| **pIF202** | VipA_WT_; Chromosomal DNA from *L. pneumophila* JR32 ([Sadosky *et al.*, 1993](#_ENREF_49)) was used as template to amplify wild-type *vipA* using oligos 1102 and 1103 and the PCR product inserted at pGEM | this work |
| **pIF300** | VipA_∆NH2_; region amplified by PCR with oligos 1112 and 1109, digested with XhoI-BamHI, and inserted at pGEM | this work |
| **pIF301** | VipA_NPY->AAA_; site-directed mutagenesis by PCR of pIF202 using oligos 1104 and 1105, followed by DpnI digestion | this work |
| **pIF302** | VipA_∆CC;_ two PCRs were performed: to amplify the NH_2_ region (oligos 1110 and 1108) and the COOH region (oligos 1111 and 1109). After digestion, respectively, with XhoI-KpnI and KpnI-BamHI, a triple ligation was made with pGEM | this work |
| **pIF303** | VipA_∆Pro_; two PCRs were performed: to amplify the NH_2_ region (oligos 1106 and 1108) and the COOH region (oligos 1107 and 1109). After digestion, respectively, with XhoI-KpnI and KpnI-BamHI, a triple ligation was made with pGEM | this work |
| **pEGFP-N1 derivatives** | **Expression of VipA-EGFP fusions in mammalian cells** | Clontech |
| pIF203 | VipA_WT_-EGFP | [Franco *et al.*, 2012](#_ENREF_15) |
| pIF304 | VipA_∆NH2_-EGFP; region removed from pIF300 by digestion with XhoI-BamHI and inserted into pEGFP-N1 | this work |
| pIF305 | VipA_NPY->AAA_-EGFP; region removed from pIF301 by digestion with XhoI-BamHI and inserted into pEGFP-N1 | this work |
| pIF306 | VipA_∆CC_-EGFP; region removed from pIF302 by digestion with XhoI-BamHI and inserted into pEGFP-N1 | this work |
| pIF307 | VipA_∆Pro_-EGFP; region removed from pIF303 by digestion with XhoI-BamHI and inserted into pEGFP-N1 | this work |
| pIF320 | VipA_NH2_-EGFP; region amplified from pIF202 by PCR with oligos 1108 and 1301, digested with XhoI-BamHI and inserted into pEGFP-N1 | this work |
| pIF321 | VipA_CC_-EGFP; region amplified from pIF202 by PCR with oligos 1112 and 1299, digested with XhoI-BamHI and inserted into pEGFP-N1 | this work |
| pIF322 | VipA_COOH_-EGFP; region amplified from pIF202 by PCR with oligos 1300 and 1109, digested with XhoI-BamHI and inserted into pEGFP-N1 | this work |
| pIF326 | VipA_∆COOH_-EGFP; region amplified from pIF202 by PCR with oligos 1108 and 1299, digested with XhoI-BamHI and inserted into pEGFP-N1 | this work |

Table S2. *Continued*

| **Plasmid** | **Purpose/Construction** | **Reference** |
| --- | --- | --- |
| **pEF6/Myc-His A derivatives** | **Expression of VipA-EGFP fusions in mammalian cells** | Life Technol. |
| pIF328 | VipA_WT_-myc; region amplified by PCR with oligos 1577 and 1580, digested with XbaI-BamHI and inserted into pEF6/Myc-His A | this work |
| pIF344 | VipA_∆NH2_-myc; region amplified by PCR with oligos 1578 and 1580, digested with XbaI-BamHI and inserted into pEF6/Myc-His A | this work |
| pIF361 | VipA_∆COOH_-myc; region amplified by PCR with oligos 1577 and 1594, digested with XbaI-BamHI and inserted into pEF6/Myc-His A | this work |
| pIF368 | VipA_∆CC_-myc; region amplified by PCR with oligos 1577 and 1580, digested with XbaI-BamHI and inserted into pEF6/Myc-His A | this work |
| **pKS84 derivatives** | **Expression of VipA-GFP fusions in *S. cerevisiae* from a galactose inducible promoter** | deFelipe *et al.*, 2008 |
| pIF206 | VipA_WT_-GFP | [Franco *et al.*, 2012](#_ENREF_15) |
| pIF308 | VipA_∆NH2_-GFP; region amplified by PCR with oligos 1192 and 1103, digested with BamHI-HindIII and inserted in the same sites of pKS84 | this work |
| pIF309 | VipA_NPY->AAA_-GFP; region amplified by PCR with oligos 1102 and 1103, digested with BamHI-HindIII and inserted in the same sites of pKS84 | this work |
| pIF310 | VipA_∆CC_-GFP; region amplified by PCR with oligos 1102 and 1103, digested with BamHI-HindIII and inserted in the same sites of pKS84 | this work |
| pIF311 | VipA_∆Pro_-GFP; region amplified by PCR with oligos 1102 and 1103, digested with BamHI-HindIII and inserted in the same sites of pKS84 | this work |
| pIF323 | VipA_NH2_-GFP; region amplified by PCR with oligos 1102 and 1298, digested with BamHI-HindIII and inserted in the same sites of pKS84 | this work |
| pIF324 | VipA_CC_-GFP; region amplified by PCR with oligos 1113 and 1296, digested with BamHI-HindIII and inserted in the same sites of pKS84 | this work |
| pIF325 | VipA_COOH_-GFP; region amplified by PCR with oligos 1297 and 1103, digested with BamHI-HindIII and inserted in the same sites of pKS84 | this work |
| pIF327 | VipA_∆COOH_-GFP; region amplified by PCR with oligos 1102 and 1296, digested with BamHI-HindIII and inserted in the same sites of pKS84 | this work |
| **pIF215 derivatives** | **Expression of VipA-mCherry fusions in *S. cerevisiae* from a galactose inducible promoter** | [Franco *et al.*, 2012](#_ENREF_15) |
| pIF216 | VipA_WT_-mCherry | [Franco *et al.*, 2012](#_ENREF_15) |
| pIF318 | VipA_∆NH2_-mCherry; DNA fragment removed from pIF308 by digestion BamHI-HindIII and subcloned at the same sites of pIF215 | this work |
| pIF319 | VipA_∆CC_-mCherry; DNA fragment removed from pIF310 by digestion BamHI-HindIII and subcloned at the same sites of pIF215 | this work |
| pIF331 | VipA_NH2_-mCherry; DNA fragment removed from pIF323 by digestion BamHI-HindIII and subcloned at the same sites of pIF215 | this work |

Table S2. *Continued.*

| **Plasmid** | **Purpose/Construction** | **Reference** |
| --- | --- | --- |
| pIF332 | VipA_CC_-mCherry; DNA fragment removed from pIF324 by digestion BamHI-HindIII and subcloned at the same sites of pIF215 | this work |
| pIF333 | VipA_COOH_-mCherry; DNA fragment removed from pIF325 by digestion BamHI-HindIII and subcloned at the same sites of pIF215 | this work |
| pIF330 | VipA_∆COOH_-mCherry; DNA fragment removed from pIF327 by digestion BamHI-HindIII and subcloned at the same sites of pIF215 | this work |
| pJB3 | VipA_NPY->AAA_-mCherry; DNA fragment removed from the pIF309 by digestion BamHI-HindIII and subcloned at the same sites of pIF215 | this work |
| pJB4 | VipA_∆Pro_-mCherry; DNA fragment removed from pIF311 by digestion BamHI-HindIII and subcloned at the same sites of pIF215 | this work |
| **pET derivatives** | **Expression and purification of His_6_-VipA proteins** | Novagen |
| pET15b-*vipA* | pET15b-His_6_-VipA_WT_ | [Franco *et al.*, 2012](#_ENREF_15) |
| pIF358 | His_6_-VipA_∆NH2_; region amplified by PCR with oligos 1592 and 1591, digested with NdeI-BamHI and inserted into pET28b | this work |
| pIF373 | His_6_-VipA_∆COOH_; region amplified by PCR with oligos 1590 and 1299, digested with NdeI-BamHI and inserted into pET28b | this work |
| pIF374 | His_6_-VipA_∆CC_; region amplified by PCR with oligos 1590 and 1591, digested with NdeI-BamHI and inserted into pET28b | this work |
| pJB9 | His_6_-VipA_COOH_; region amplified by PCR with oligos 1775 and 1591, digested with NdeI-BamHI and inserted into pET28b | this work |

Table S3. Oligonucleotides used in this work

| **Oligonucleotide** | | **Sequence (5’→3’)*^a^*** | | |
| --- | --- | --- | --- | --- |
| 1102 | AAAAGGATCCATGCCTATCAGTAATG | | |  |
| 1103 | GGGGAAGCTTGAGATTTTTTTTTTCG | | |  |
| 1104 | TCAGCAAAGACCGCTGCCGCCTTACTCGATTG | | |  |
| 1105 | CAATCGAGTAAGGCGGCAGCGGTCTTTGCTGA | | |  |
| 1106 | AAAGGTACCCTCTTGACTGATTGC | | |  |
| 1107 | AAAGGTACCAATGATACGGATGGTCAAGC | | |  |
| 1108 | ACTCGAGCGCCACCATGC | | |  |
| 1109 | ATTCGCCCTGGATCCCGGAG | | |  |
| 1110 | CCCGGTACCCTTTTGAGCTAAAATAAAC | | |  |
| 1111 | AAAGGTACCACAAATGCCAAACCC | | |  |
| 1112 | ACTCGAGCGCCACCATGTTTATTTTAGCTCAAAAGG | | |  |
| 1113 | AAAAGGATCCATGTTTATTTTAGCTCAAAAGGC | | |  |
| 1192 | AAAAGGATCCATGTTTATTTTAGCTCAAAAG | | |  |
| 1296 | GTTTAAGCTTTGTGATTTCGCTTAGAG | | |  |
| 1297 | TCTAGGATCCATGACAAATGCCAAACCC | | |  |
| 1298 | GAGCAAGCTTAAACGCTTGTTGCGTC | | |  |
| 1299 | TGGGTGGATCCTTTGTGATTTCGCTTAGAG | | |  |
| 1300 | ACTCGAGCGCCACCATGACAAATGCCAAACCCAAAATAGAAAC | | |  |
| 1301 | TTTGAGGATCCATAAACGCTTGTTGCGTC | | |  |
| 1577 | AAAAAAGGATCCACCATGGCGCCTATCAGTAATGCC | | |  |
| 1578 | AAAAAAGGATCCACCATGGCGTTTATTTTAGCTCAAAAGG | | |  |
| 1580 | GGGGGGTCTAGAGAGATTTTTTTTTTCGACGGG | | |  |
| 1581 | AAAAAATCTAGAGAAAACGCTTGTTGCGTCATGACC | | |  |
| 1594 | AAAAAATCTAGAGATGATTTCGCTTAGAGTTTG | | |  |
| 1590 | AGACATATGATGCCTATCAGTAATGCCTTT | | |  |
| 1591 | AGAGGATCCCTAGAGATTTTTTTTTTCGACGGG | | |  |
| 1592 | GCAACATATGTTTATTTTAGCTCAAAAGGCC | | |  |
| 1579 | AAAAAAGGATCCACCATGGCGACAAATGCCAAACCCAAAATAG | | |  |
| 1582 | | | AAAAAATCTAGAGATGTGATTTCGCTTAGAGTTTG |  |
| 1775 | | | CTAAGCCATATGACAAATGCCAAACCCAAAATAGAAAC |  |

*^a^* Restriction sites are underlined.


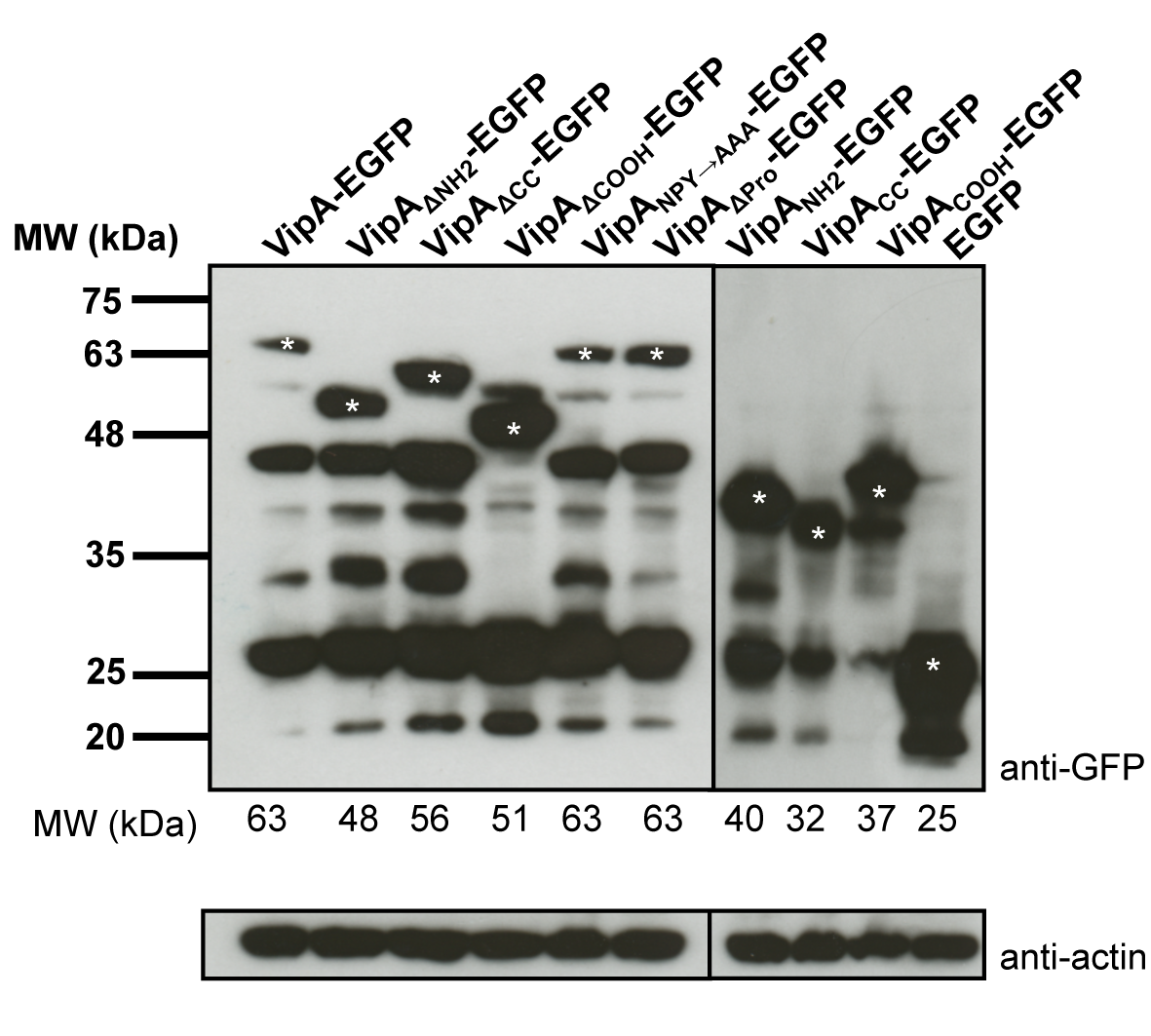


**Figure S1. Accumulation of VipA-EGFP fusion proteins in transiently transfected CHO cells.**

CHO cells were transfected for 24 h with plasmids encoding the indicated proteins. The cells were then lysed and analyzed by immunoblotting with anti-GFP and anti-actin (loading control) antibodies. The values below the immunoblot image indicate the predicted molecular mass in (kDa) of each of the proteins analyzed (marked with an asterisk in the blot image).


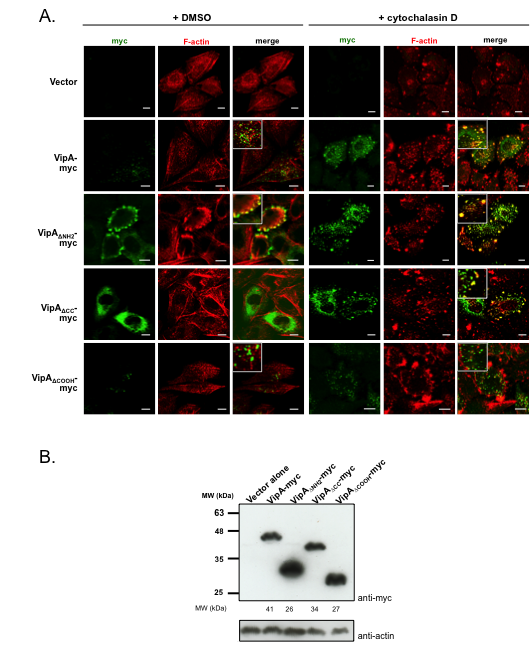


**Figure S2.** **Localization and accumulation of VipA-myc fusion proteins in mammalian CHO cells.**

CHO cells were transfected for 24 h with plasmids encoding the indicated proteins or the empy vector (pEF6-Myc/His A; Life Technologies) and analyzed by immunofluorescence microscopy (A) or immunoblotting (B).

A. After transfection, cells were incubated with DMSO (left panel) or 10 μM cytochalasin D for 30 min (right panel) immediately before fixation and immunolabeling of VipA proteins with an anti-myc antibody and an appropriate fluorophore-conjugated antibody and staining of F-actin with phalloidin-Alexa 555. Images were collected in a Zeiss LSM710 confocal microscope. Scale bars, 10 μm.

B. After transfection, cells were lysed and analyzed by immunoblotting with anti-myc and anti-actin (loading control) antibodies. The values below the immunoblot image indicate the predicted molecular mass in (kDa) of each of the proteins analyzed.


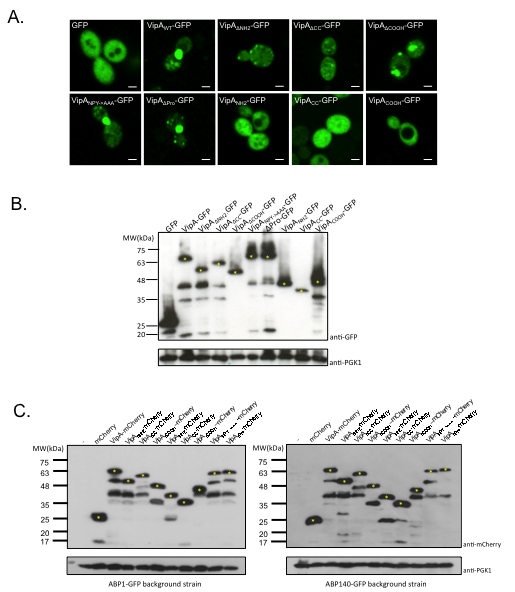


**Figure S3.** **Subcellular localization and accumulation of VipA-GFP proteins in *S. cerevisiae.***

A. *S. cerevisiae* NSY01-derived strains carrying VipA-GFP fusions used in the Vps assays (Fig. 6) were grown in the presence of galactose and live cells were visualized on a Zeiss LSM710 confocal microscope. Scale bars, 2 μm.

B. *S. cerevisiae* NSY01-derived strains carrying VipA-GFP fusions grown as described in A were lysed and equivalent amounts of cell lysates were analyzed by immunoblotting using an anti-GFP or anti-PGK1 (3-phosphoglycerate kinase; loading control) antibodies. The asterisk marks the bands corresponding to VipA-GFP fusion proteins.

C. *S. cerevisiae* THY157 or YCY027-derived strains (ABP1-GFP or ABP140-GFP) carrying VipA-mCherry

fusions grown as described in A were lysed and equivalent amounts of cell lysates were analyzed by immunoblotting using an anti-mCherry or anti-PGK1 (loading control) antibodies. The asterisk marks the bands corresponding to VipA-mCherry fusion proteins.
